# Biomedical Applications of Microfluidic Devices: A Review

**DOI:** 10.3390/bios12111023

**Published:** 2022-11-16

**Authors:** Ghazaleh Gharib, İsmail Bütün, Zülâl Muganlı, Gül Kozalak, İlayda Namlı, Seyedali Seyedmirzaei Sarraf, Vahid Ebrahimpour Ahmadi, Erçil Toyran, Andre J. van Wijnen, Ali Koşar

**Affiliations:** 1 Faculty of Engineering and Natural Science, Sabanci University, Istanbul 34956, Turkey; 2 Sabanci University Nanotechnology Research and Application Centre (SUNUM), Istanbul 34956, Turkey; 3 Center of Excellence for Functional Surfaces and Interfaces for Nano Diagnostics (EFSUN), Faculty of Engineering and Natural Sciences, Sabanci University, Istanbul 34956, Turkey; 4 Department of Biochemistry, University of Vermont, 89 Beaumont Avenue, Burlington, VT 05405, USA; 5 Turkish Academy of Sciences (TÜBA), Çankaya, Ankara 06700, Turkey

**Keywords:** micromixers, particle separation, cell sorting, particle enrichment, electrophoresis, dielectrophoresis, magnetophoresis, acoustophoresis, pressure fields, thermal fields, optical trapping, disease modeling, biomedical applications, lab-on-a-chip, organ-on-a-chip, point-of-care, cancer diagnosis, biosensors

## Abstract

Both passive and active microfluidic chips are used in many biomedical and chemical applications to support fluid mixing, particle manipulations, and signal detection. Passive microfluidic devices are geometry-dependent, and their uses are rather limited. Active microfluidic devices include sensors or detectors that transduce chemical, biological, and physical changes into electrical or optical signals. Also, they are transduction devices that detect biological and chemical changes in biomedical applications, and they are highly versatile microfluidic tools for disease diagnosis and organ modeling. This review provides a comprehensive overview of the significant advances that have been made in the development of microfluidics devices. We will discuss the function of microfluidic devices as micromixers or as sorters of cells and substances (e.g., microfiltration, flow or displacement, and trapping). Microfluidic devices are fabricated using a range of techniques, including molding, etching, three-dimensional printing, and nanofabrication. Their broad utility lies in the detection of diagnostic biomarkers and organ-on-chip approaches that permit disease modeling in cancer, as well as uses in neurological, cardiovascular, hepatic, and pulmonary diseases. Biosensor applications allow for point-of-care testing, using assays based on enzymes, nanozymes, antibodies, or nucleic acids (DNA or RNA). An anticipated development in the field includes the optimization of techniques for the fabrication of microfluidic devices using biocompatible materials. These developments will increase biomedical versatility, reduce diagnostic costs, and accelerate diagnosis time of microfluidics technology.

## 1. Introduction

Recent advances in the design and development of microfluidics (MFs) devices have made it possible to miniaturize conventional biochemical laboratory protocols into a microchannel networking system, which has emerged as an efficient and cost-effective tool. Biomedical microdevices include integrated structures consisting of numerous micro- and nano-sized integrated devices, where many processes from particle manipulation to sensing take place in the platform. Although different types of microfluidic devices can perform similar tasks in biomedical applications, passive microfluidic systems are mainly used for particle manipulation [1,2,3,4,5] and mixing liquids [4,6], while active types contribute more to particle trapping [7,8,9,10,11,12,13,14,15,16,17,18,19] and sensing [20,21,22,23,24]. Passive devices are governed by diffusion, inertial forces, secondary flows, and geometry-induced turbulence and particle manipulation; active microfluidic devices generate streams depending on external energy to disturb particles or fluids inside microfluidic devices. Depending on the geometric design, mixing ratios of fluids could be relatively high, and separation of particles, which have different sizes and densities under the influence of internal forces, can reach high-efficiency values in microfluidic channels. External forces due to acoustic pressure fields, electric fields, magnetic fields, thermal fields, pressure fields, and optical fields could manipulate biological or chemical particles and mix fluids in biomedical applications. In addition, some active manipulation techniques with functional surfaces coated on the transduction area can also sense some unique biological structures, such as DNAs and biomarkers. Briefly, passive MFs, where internal forces are effective, and active MFs, which perform operations under the influence of external forces, are two categories regarding microfluidic devices. There are several biomedical applications in microfluidics devices. One of the most promising applications of microfluidics in biomedical sciences is the diagnosis of diseases, including cancer diagnosis and infectious diseases. However, further development of microfabrication permits employment of microfluidics devices in disease modeling, tissue engineering, and organ-on-a-chip. Moreover, microfluidics–biosensing technology has become popular for applications such as point-of-care testing, biosensors, and cell manipulations [25]. This narrative review discusses recent advancements in microfluidics systems and their relevant biomedical applications. The table of content is available in the Supplementary Material.

## 2. Microfluidics

Microfluidic devices are generally categorized into passive and active devices. Although the effect of internal forces, diffusion, and secondary flows are very effective in fluid mixing and particle manipulation, they operate within geometry-dependent limits in passive/inertial microfluidic devices. In active microfluidic devices, the restriction involves the interaction between the target and external energy source in contrast to passive microfluidic chips. For example, magnetic fields can only affect structures with magnetic properties. Even though there is a limit between the energy source and target structure, some energy sources, such as acoustic fields and pressure fields, eliminate manipulation limits, and many biomedical applications benefit from the incorporation of microfluidic devices over time. This section discusses the most recent studies of microfluidics and their outputs, focusing on passive and active devices as the major distinction.

### 2.1. Passive Microfluidics

The main applications of passive microfluidic devices are fluid mixing, particle focusing, separation, sorting, and isolation, which are all manipulation techniques of fluids and particles for specific applications. Even though geometrical limits reduce the scope of applications, some biomedical implementations of passive microfluidic devices (e.g., organ-on-a-chip) open new horizons that overcome this limit.

#### 2.1.1. Inertial Micromixers

**Figure 1:**
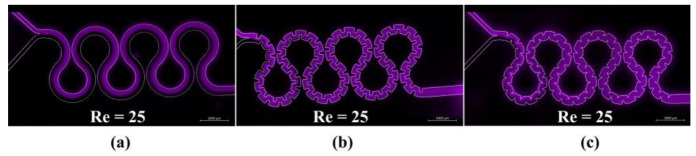
The fluorescence intensity maps of micromixer M1 (**a**), M3 (**b**), and M7 (**c**) at Re = 25 [6]. Copyright 2021, Elsevier.

Passive micromixers with wide applications from chemical reactions to biological analysis processes rely on their channel geometry. These low costs and complexity are based on Dean vortices, or secondary flow, seen in curved microchannels to improve the mixing efficiency [4]. In addition to chaotic advection mixing generated by curved microchannels, designing different-shaped baffles on channel walls can further enhance the mixing index by constantly fluid folding and stretching. Ahmadi et al. [6] experimentally and numerically compared six novel-designed microchannels (i.e., M2 to M6) with a previously studied serpentine micromixer (M1). Among the designed microchannels, M3 and M7 could provide high mixing indexes of 98% at Reynolds numbers (Re) of 20 and 35, respectively, implying mixing performance can be enhanced even at very low Re by designed baffles on channel walls (Figure 1).

#### 2.1.2. Sorting, Separation, and Isolation

Principal areas of interest in biomedical research are the analysis of particular cell types in samples containing various components by purification and sorting. Depending on the application, target cell types could have moderate or low populations inside the primary sample [26]. Considering that microfluidic devices are potent platforms for manipulating particles inside microchannels, lab-on-a-chip platforms have been developed for cell sorting, separation, or isolation based on the biological or physical properties of the target cells. Passive microfluidics, which operate independently of any external force, are based on different mechanisms, including microfiltration, inertial and secondary flow, deterministic lateral displacement (DLD), and pinch flow fractionation (PFF) [26,27,28].

(a) Microfiltration

**Figure 2:**
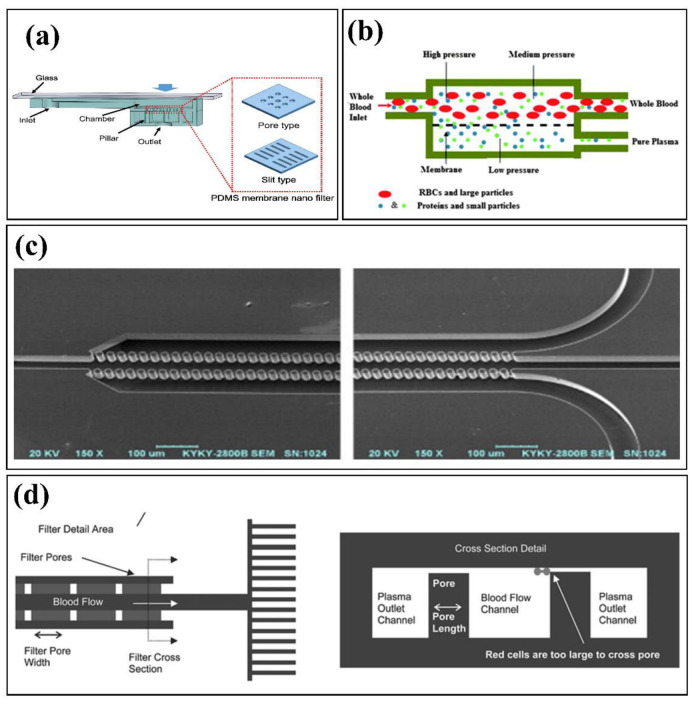
Microfiltration examples: (**a**) Dead-end membrane-based filtration, adopted with permission from [29], Copyright 2019, Elsevier; (**b**) Cross-flow membrane-based filtration, adopted with permission from [33] Copyright 2011, Royal Society of Chemistry; (**c**) Cross-flow pillar-based filtration, adopted with permission from [30], Copyright 2008, Elsevier; and (**d**) Cross-flow weir-based filtration, adopted with permission from [31], Copyright 2005, Royal Society of Chemistry.

This technique employs the size of floating components inside the original sample to trap the particles in specially designed restrictive elements. Considering the type of elements used for separation (Figure 2), filtration is categorized into three main groups, including membranes [29], pillars [30], and other flow barriers (e.g., weirs) [31]. In addition, based on the flow direction through micro-posts or along them, microfiltration platforms are classified into dead-end [32] and crossflow [33] types. the efficiency of capturing large particles in dead-end type is better than that of crossflow filtration. However, the latter type mitigates the clogging problem [34].

(b) Inertial Focusing and Secondary Flows

**Figure 3:**
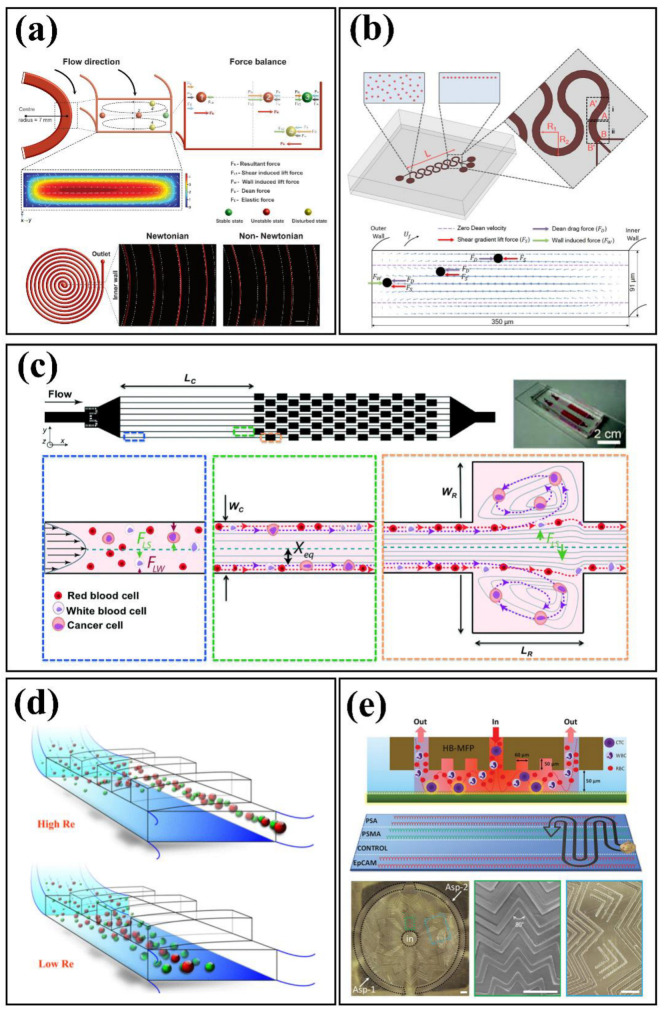
Inertial and secondary flow examples: (**a**) viscoelastic non-Newtonian spiral device, reprinted with permission from [44], Copyright 2021, Springer Nature; (**b**) serpentine device, reprinted with permission from [1], Copyright 2016, Springer Nature; (**c**) successive contraction and extraction channels, reprinted with permission from [38], Copyright 2013, Royal Society of Chemistry; (**d**) top surface slanted grooves configuration, reprinted with permission from [39], Copyright 2017, IEEE; and (**e**) Herringbone structure, reprinted with permission from [40], Copyright 2021, Wiley-VCH GmbH.

Under laminar flow conditions and the absence of any external forces, suspended particles travel among fluid streamlines until an equilibrium is reached. The latter is achieved because the inertial effects become dominant under such conditions, so that the reciprocal effect of shear-induced lift and wall-induced forces on particles steers them towards the equilibrium position. For example, Segre and Silberberg experimentally observed this radial migration of neutrally buoyant solid particles in Poiseuille flow [35]. Subsequently, Di Carlo et al. [36] studied inertial focusing in straight and curved microchannels. For curved channels, in addition to induced lift forces, due to the centrifugal force, a drag force is exerted on particles by the formation of a secondary flow called the Dean flow, which is perpendicular to the primary flow. Aside from curved microchannels, other secondary flow generating geometries including spiral channels [37], serpentine channels [1], successive contraction and extraction channels [3,38], top surface slanted grooves [5,39], and herringbones structures [40] have been studied for particle sorting. The inherent high volumetric flow rates in inertial microfluidic devices make it favorable for cell separation [41]. However, the behavior of cells differs from solid particles regarding the presence of an extra deformability-induced lift force, which is driving them away from the walls [42]. Recently, inertial microfluidic platforms based on viscoelastic fluids have shown promising results in precisely focusing and manipulating particles [43]. Kumar et al. [44] investigated particles focusing in spiral channels at higher flow rates compared to previously reported values using a non-Newtonian viscoelastic fluid. The utilization of viscoelastic fluids exerts a new elastic force on particles shifting the equilibrium position from the inner wall of the curved channel to the outer wall, which is helpful for cytometry applications (Figure 3).

(c) Deterministic Lateral Displacement

Deterministic lateral displacement (DLD) microfluidic platforms offer a novel configuration and were proposed by Huang et al. [45] for continuous particle separation at low Reynolds numbers under the laminar flow conditions. This kind of device houses periodic arrays of micro-posts such that a certain distance shifts each row compared to the previous row, which generates curvy streamlines. Within this kind of microfluidic device, particles smaller than a critical value follow the streamline, whereas larger particles laterally shift between the streamlines with a defined angle. Thus, particles follow different trajectories based on size, deformability, and shape.

**Figure 4:**
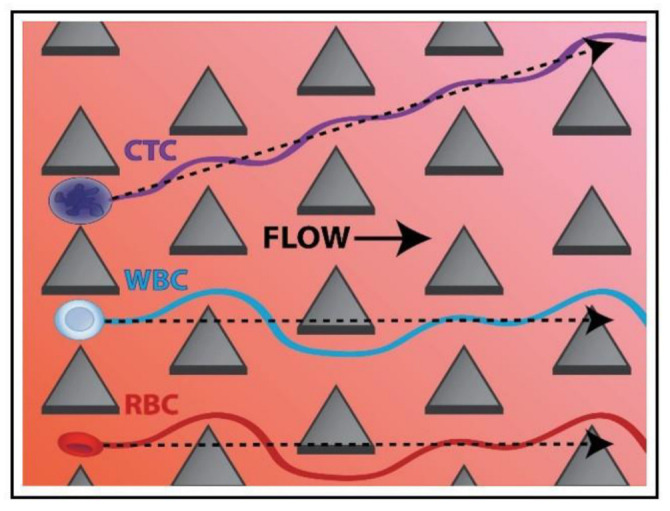
CTCs isolation based on DLD technique with triangular micro-posts, reprinted with permission from [46], Copyright 2012, AIP Publishing LLC.

DLD devices using circular micro-pillars are prone to clogging due to the formation of stagnation zones above micro-posts [26]. Circular posts are prone to generate stagnation points in which cells are trapped causing system clogging. To avoid this problem, different geometries have been investigated. As an instance, Loutherback et al. [46] reported the isolation of viable circulating tumor cells (CTCs) from blood using arrays of triangular posts (Figure 4).

(d) Pinch Flow Fractionation

Yamada et al. [47] first proposed the separation of different size particles suspended in a laminar flow with pinch flow fractionation (PFF). In this method, two fluid streams containing a liquid with suspended particles and a sheath flow enter separately through a Y-junction to a microfluidic system and are combined in a third narrow pinched segment. The flow rate ratio of the inlet flows must be such that particles are pushed to the wall of the narrow segment by the sheath flow. Therefore, the center of mass of particles differs based on their size, which steers them through different streamlines. To enhance the separation efficiency following the pinched segment, a wider segment is used to increase the lateral distance of streamlines in pinched segment. In addition, to have an optimum design, several parameters such as the total flow rate, ratio of the inlet flow rates, channel width of the pinched segment, angles of boundaries, and outlet configuration should be considered [26]. In this regard, several studies have investigated several different outlet configurations to separate particles, including symmetric [47] or asymmetric [48] channels, as well as channels equipped with micro-valves [49] and other drainage methods. Also, the PFF method could be utilized alongside other passive methods such as sedimentation PFF [50], inertial-enhanced PFF [51], and elasto-inertial PFF [52] approaches to further enhance the efficiency.

#### 2.1.3. Droplet Microfluidics

**Figure 5:**
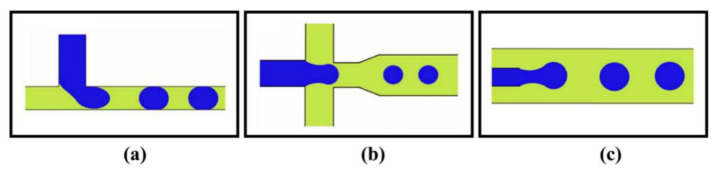
Schematic design of different droplet generation geometries: (**a**) Crossflow, (**b**) Flow-focusing, and (**c**) Co-flow; adopted with permission from [53], Copyright 2022, IOP Publishing Ltd.

The precise creation and manipulation of predefined discrete droplet volumes as immiscible liquids in microfluidic platforms provide the potential for achieving high throughput with controllable droplet properties. As a result, droplet microfluidics allows applications in different fields, including microbiology, single cell analysis, cell culture, drug delivery, micro/nano particle production, and emulsification [53,54,55,56]. In microfluidic devices, the interfacial instability of continuous and dispersed immiscible phases in oil-in-water or water-in-oil liquids results in droplet formation. By employing both passive and active techniques, these platforms offer monodisperse droplet production with high throughput and higher efficiency than traditional methods [53]. In the passive approach, where no external forces are applied in the system, the channel’s geometrical design is the most influential parameter on droplet characteristics [53]. In addition, parameters such as rheological properties of dispersed and continuous liquids and their flow rate ratios, as well as water/oil interfacial tension modification by surfactants, have been studied to attain favorable droplet creation modes [53,55]. Based on the channel configuration, passive droplet microfluidic platforms are categorized into crossflow, co-flow, and flow-focusing geometries (Figure 5) [53,55]. In a crossflow type, two immiscible liquids are introduced to the system through a Y-shape or a T-shape junction. This design requires small space on the device in comparison to other geometries and could be readily integrated into other microfluidic components [57]. Within this configuration, the produced droplets normally have plug-like shapes, but an increase in the capillary number and a decrease in the flow rate ratio makes it possible to yield spherical droplets as well [53]. Flow-focusing design is appropriate for generating high-throughput smaller spherical droplets having a minimal contact with walls compared to cross-flow configuration [55,58]. As depicted in Figure 5, the flow-focusing configuration consists of two side channels supplying the continuous flow to gird the dispersed flow streaming in the middle of the constriction channel. On the other hand, features of produced droplets in a co-flow geometry are comparable with the flow-focusing configuration with an extra capability of adjusting the droplet size by modifying the channel width of the dispersed flow [56]. The co-flow type platforms have two concentric channels providing the dispersed flow in the middle surrounded by the continuous flow, where the droplet breakup occurs at the end tip of the dispersed channel due to existing shear forces [58,59].

(a) Microfluidic-Based Materials Production

**Figure 6:**
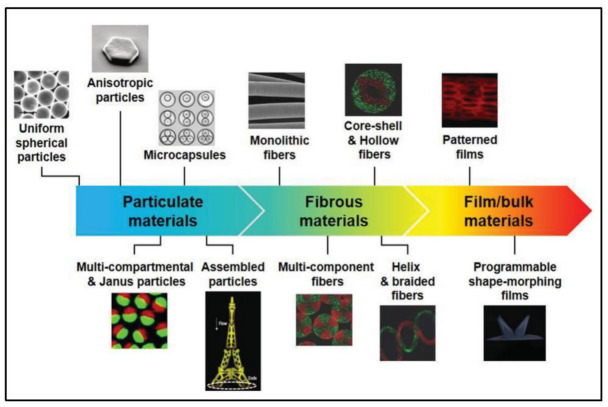
Synthesized microfluidic-based 0D/1D/2D/3D micro and nano materials. Reproduced with permission [60]. Copyright 2020, John Wiley & Sons.

The behavior of fluids inside microfluidic devices is predominantly affected by interfacial parameters rather than gravity in microscale. Therefore, by designing different geometries and changing the operating conditions, it is possible to control fluid dynamics at the microscale level. Because of this feature, microfluidics have gained traction in the production of micro and nanomaterials for biomedical applications in different fields such as diagnostics, drug delivery, organs-on-chip, tissue engineering, and stimuli-responsive biodevices [60,61]. Microfluidic devices enable researchers to control the geometry, structure, and composition of the synthesized microfluidic-based micro/nano particles, fibers, films, and bulk materials. A common technique for preparing micro/nanoparticles using microfluidics typically involves two steps [62]: forming precursor droplets of uniform size, followed by solidification of droplets using different methods such as photopolymerization [63], heating and thermal curing [64,65], solvent evaporation [66], and ionic or chemical cross-linking [67,68]. Similarly, spinning micro/nanofibers through microfluidic devices comprising different fluids’ coaxial streams is accompanied by a curing step for the central or outer precursor stream [60,61]. In addition, incorporating microfluidic nozzles into three-dimensional printers allows precise control over the spatial distribution of fed inks to form heterogeneous compositions of two-dimensional films and customized three-dimensional structures [60] (Figure 6).

### 2.2. Active Microfluidic Devices

**Figure 7:**
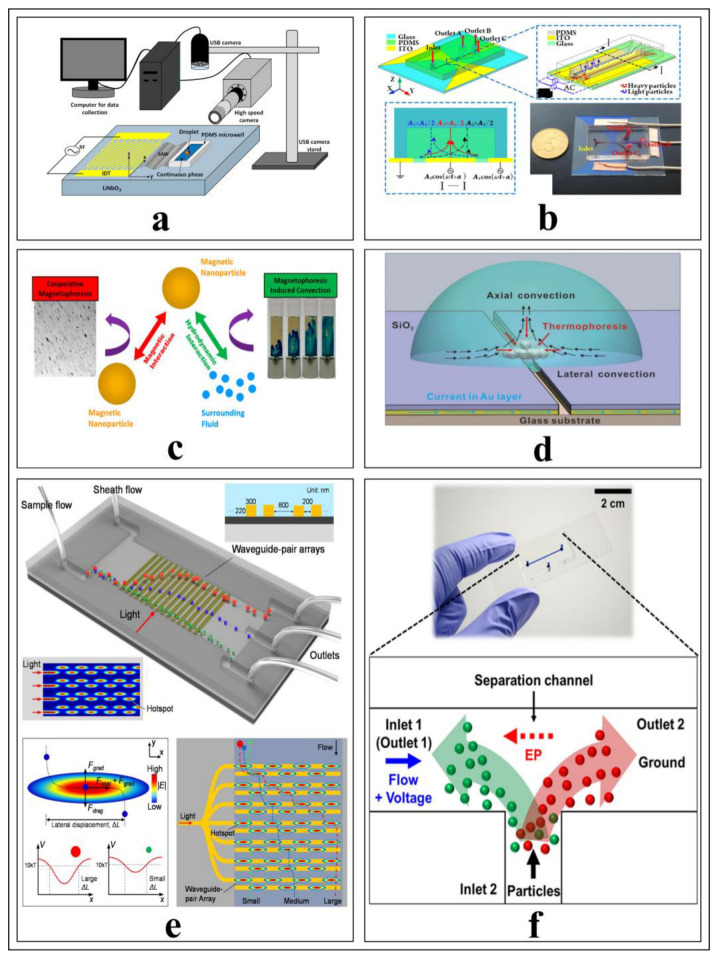
Microfluid techniques: (**a**) acoustic radiation force and experimental setup [18], Copyright 2022, Langmuir; (**b**) Electrophoresis and dielectrophoresis configurations in microfluidic devices [10], Copyright 2019, American Chemical Society; (**c**) Manipulation of magnetic nanoparticles using Magnetophoresis method [70], Copyright 2020, Langmuir; (**d**) Visualization of thermal field particle manipulation in a droplet [12], Copyright 2016, Scientific Reports; (**e**) Optical manipulation of particles inside a microfluidic channel under a certain flow rate [8], Copyright 2021, Sensors and Actuators B: Chemical; and (**f**) Manipulation of particles by driving pressure field with electric field [71], Copyright 2016, Scientific Reports.

Active microfluidic devices are influential small size platforms, where flow regimes, stationary droplets, or particles inside the fluid are manipulated for specific purposes under the influence of external forces (Figure 7), such as acoustic pressure fields, electric fields, magnetic fields, optical or electromagnetic fields, and thermal gradients [69]. Regardless of the characteristics of microfluidic devices, the type of transducer that generates the external force is limited by the sample properties used for biomedical applications. To exemplify, almost all micro and nanoparticles can change motions with the direct effects of acoustic-based transducers, but the electrical or magnetic properties of the particles highly depend on whether the source is an electric field or a magnetic field. Active microfluidic devices have found many applications and are currently being developed more and more with time. They could be categorized depending on the external energy type, and an external energy source is selected according to the electrical, magnetic, and physical properties of the target particle or liquid. The difficulty here is that, except for the acoustic and pressure field effective devices, they are selected according to the characteristics of the target, and their design is dependent on it. Acoustic resonators with microfluidic devices can manipulate almost any type of particle, but submicron particles require high-frequency signals, may exceed 1 GHz, and may require nanostructures to transduce the electric signal into acoustic waves. Electric and magnetic fields are more successful in affecting submicron particles and liquids than acoustic waves. The focused laser beam can drag particles with high radiation pressure in optical methods. Although there is independence from the characteristics of the target in pressure-effect systems, vibrating physical mechanisms can direct the particles with specific criteria. Finally, manipulating particles under the thermal gradient effect is possible to a certain extent.

#### 2.2.1. Dynamic Micromixers

**Figure 8:**
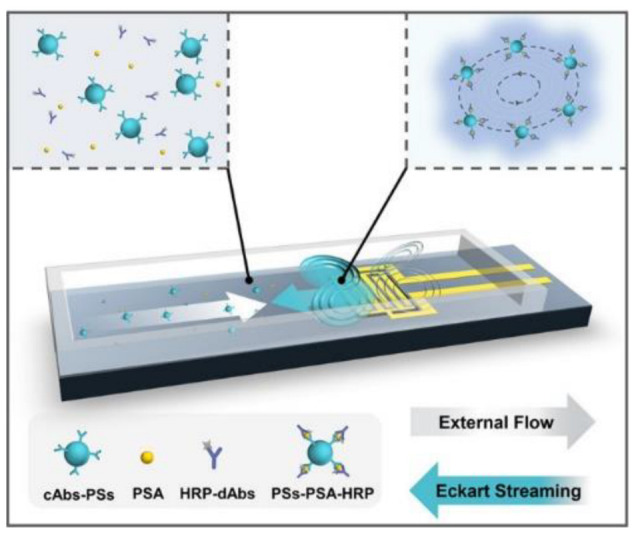
A simple view of an active micromixer device [73], Copyright 2021, ACS Publications.

The working principle of micromixers is based on two main effects: molecular interdiffusion with hydrostatic potential and turbulence formation inside a microchannel by the effect of geometrical shapes of microchannels or the external energy sources causing chaotic advection [72]. Induction of mixing processes inside microfluidic channels using external energy sources is realized in active or dynamic micromixers, which is schematized in Figure 8. Unlike inertial microfluidic channels, the energy source of dynamic manipulators could be an acoustic field, electric field, magnetic field, thermal gradient, and externally stimulated micro-mechanisms inside a microchannel.

(a) Acoustic Field-Driven Micromixers

Fluids can be mixed with high efficiency by creating acoustic streaming, which perturbs the fluid flow [74]. According to Nam’s study, 100% mixing efficiency can be achieved by creating three-dimensional acoustic-fluid interactions in the microchannel [75]. Surface acoustic wave manipulators with a 30 MHZ resonance frequency were produced by designing dual LiNbO3-based Focused Interdigitated Transducer (F-IDT), one for the bottom side of the channel and the other for the top of the microchannel. Thus, when the Surface Acoustic Waves (SAWs) advancing from both surfaces interact with deionized water and fluorescent particles, they are a strong vortex that leads to high-speed mixing in the channel at 4.44 ≤ Re ≤ 22.22 [75]. Blockage of channels, nanoprecipitation of particles, particle sizes, and batch-to-batch variation are common problems in nanoparticle synthesis. To overcome these obstacles, Rasouli & Tabrizian proposed to shape sharp edges within the microchannel and create vortices by vibrating the bubbles with strong acoustic energy using a PZT (lead zirconate titanate) disc driven in several kHz ranges [76]. Chemical and biological mixtures could reach a mixing efficiency (MI) as high as 80% within milliseconds (e.g., 0.8 ms), while obtaining homogeneous and non-precipitating mixtures [76]. It seems challenging to achieve both rapid and high efficiency mixing with a single device. Yet, Bachman et al. [77] examined the effectiveness of the mixture by adjusting the flow rate. They observed that varying flow rates from 20–2000 uL.min^−1^ is very effective in obtaining a homogenous solution in the outlet, even though the mixing index is not as high as desired (maximum < 0.5) [77]. Because mixing is complicated in linear microchannels, mixing efficiencies up to 90% can be achieved at specific flow rates by integrating different micro geometries (e.g., domed structures) into linear channels [77]. Although it occurs at low flow rates, a high mixing efficiency could be achieved at a 39.6 MHZ and 20 V signal amplitude. Due to the acoustic stream created by the F-IDTs placed around the dome, effective mixing could be reached [78]. Instead of using SAW, bulk acoustic waves could be more advantageous for mixing. A star-shaped micro-oscillator was driven by bulk acoustic waves, allowing a 91% efficiency within 4.1 ms [79]. Fluid mixing is also convenient for diagnosis or detection systems developed as portable point-of-care (POC) devices. However, the amount of fluid used in such procedures is not acceptable for clinical applications. Acoustic waves generated by linear IDTs driven with a resonance frequency of 390 MHZ trap the fluorescent polystyrene particles diluted with PBS and mixed with immunocomplexes by the acoustic stream [73]. In a prostate-specific antigen (PSA) detection system, a wide dynamic response range from 0.3 ng/mL to 10 ng/mL was obtained with a detection limit of 0.2 ng/mL in a 10 µL sample [73]. In addition to the mixture of liquids in a continuous flow, mixtures in the form of droplets are also frequently used methods in biological applications. Piezoelectric transducers, based on PZT with a signal of several hundred kHz based on PZT, have a higher mixing efficiency and, provide rapid mixing by creating acoustic flow in the droplet [80]. Rounded piezoelectric transducers operate at low frequencies but generate highly effective acoustic waves. Still, SAW-based transducers operating at higher frequencies are frequently used because they can be produced in different geometric shapes. Acoustophoretic forces cause acoustic streams that perturb tiny droplets to rapid mixing and result in detectable (by photodiodes) color changes within a short time [81].

(b) Electric Field-Driven Micromixers

The conductivity feature is not sought in biological or chemical samples for acoustic field micromixers. However, electrophoresis, dielectrophoresis, and magnetophoresis are manipulation techniques that need to be developed, depending on particle properties such as the conductivity, magnetization, and dipole moment. Although there are many obstacles, the experiments with different parameters such as frequency, flow rate, and solution conductivity, the mixing efficiency exceeded 90% when the following parameters were used: flow rate is 0.728 µL/min, the electrode conductivity is 0.2 S/m (1 S/m), and the applied voltage of 52.5 $V_{p-p}\;$with 1 MHz oscillating frequency [82]. Mixing of fluorescently labeled versus dye-free KCl solutions with the same conductivities could be mixed with high efficiency (94.7%) using the charge-induced electroosmosis flow (ICEO) created by applying a signal of 14 V at 400 MHz to ITO electrodes [83]. In another study, AC electroosmosis was implemented at nanoscales for lipid-based drug delivery via vesicles using nanoprecipitation with a phase-controlled field-effect micromixer and three-fingered sinusoidal shaped and linear electrodes [84,85]. These unique phase-based mixing systems permit high mixing efficiencies over 90% at a volumetric flow rate of 4 µL/min, corresponding to ~13.9 mm/s under optimized voltage excitation conditions [84,85]. As a droplet-based micromixer platform, AC electrostatic excited micromixer is a unique microfluidic system used to induce vibration and deformation in a liquid marble, but it could have negative consequences of high electric field strengths of 385 kV.m^−1^ in biological applications [86]. On the other hand, the lab-on-a-foil concept is the AC electroosmosis micromixer that emerged as an innovative approach [87]. In the study, tooth-shaped planar electrodes were fabricated inside the PDMS microchannel, and electroosmotic flow (EOF) was investigated depending on the flow rate and applied signal frequency [87]. According to the results, the optimum values for mixing performance were 1 Hz for signal frequency and 15, 20, 25, and 30 μL/min for flow rates [87].

(c) Magnetic Field-Driven Micromixers

Similar to electrophoresis, magnetophoretic micromixers developed according to material properties are also highly effective for magnetofluids and magnetic particles. Rapid mixing of deionized water with Fe_3_O_4_ ferrofluid in a Y-shaped microchannel, integrated into a permanent magnet, has been studied for the permanent magnets [88]. A magnetic field of 3000 G, which was applied to the magnetic nanoparticles flowing inside a linear microfluidic channel, shortened the mixing length and increase the mixing efficiency to 95% within less than two seconds [88]. For the same purpose, numerical and experimental results of an integrated magnetic micromixer design with a Y-shaped microchannel and a uniform magnetic field were shared to obtain rapid mixing of ferrofluid and deionized water. The mixing performance of the micromixer could be provided and optimized by adding microwires to the structure [89]. As a result, the mixing efficiency could reach around 99.06% [89]. Rotating magnets were used in mixing and cell lysis. The magnetic slabs were attached to a rotating circular disk as magnetic stirrers, changing angular velocities from 0 to 480 rpm to investigate the shaking effect on mixing the DI-water with glycerol 75%. As a result of high mixing efficiency and production of viscous stresses, this platform was successfully implemented for cell lysis [90]. The following study presented a magnetofluid mixer for rapid mixing of ferrofluid and distilled water without producing harmful Joule heat up, when considering the microfluidic flow rate, magnet placement angle, and magnet dimensions. The results were able to increase the mixing performance of the magnetofluidic micromixer up to 50% with the optimization of magnet properties [91]. Unlike static magnets, electromagnets driven by direct current (DC), or alternating current (AC) are also widely used in mixing processes. By adjusting the desired wire cross-section and dimensions, the desired magnetic field magnitude could be created statically or dynamically, and rapid and high-efficiency mixing of chemical or biological microfluidic samples with magnetic properties could be achieved [92,93,94,95].

(d) Thermal Field Micromixers

Temperature gradients originating from temperature differences between two points allows masses to be manipulated at certain rates and adapting the methodology in various applications. The main advantage for these micromixers is the use of very low AC signals (1–30$\;V_{p-p}$) with frequencies from hundreds of kHz to several megahertz (MHz) capable of eliminating Faradaic currents and associated reaction air bubbles [96]. This methodology relies on the Marangoni effect, which involves temperature–gradient dependent mass transport across the interface of two fluid surfaces with different temperatures by thermo-capillary convection. By focusing the laser on plasmonic metal nanostructures placed in a microchannel, a temperature of 200 °C could be reached with local light–heat conversion, and bubbles with a diameter of 10 µm could be formed, allowing the solution to be mixed in the microchannel [97].

(e) Pressure Field Micromixers

Effective fluid mixing can also be achieved by pressure fields that cause pulsatile micromixing, which are created by oscillatory micropumps, or mechanical parts integrated into the microchannel. The oscillation unit controlled by the switching frequency generates pressure fields in the microchannel, and mixing is achieved momentarily. According to different flow rates and oscillation frequencies, the studies on various fluids reported high mixing efficiencies from 75% to 99%, proving the potential of pulsed field-effect micromixers [98,99,100,101,102].

#### 2.2.2. Particle Separation

**Figure 9:**
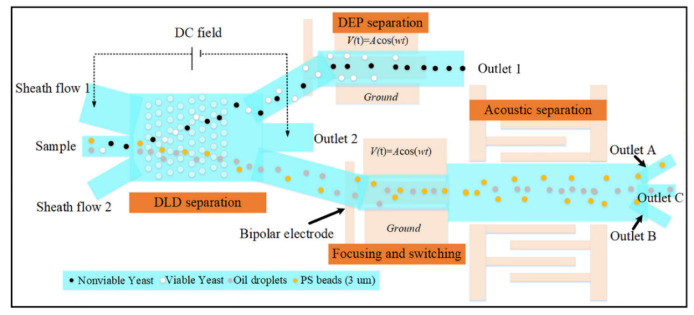
A cascade system to schematize integrated active type particle separation modules [103], Copyright 2021, ACS Publications.

Particle separation represents a process that goes beyond the mere separation of two types of micro/nanoparticles, schematized in Figure 9. Rather, this process includes precise and critical applications such as the purification of submicron particles for chemical purposes or the separation of cancerous cells from healthy cells in biomedical studies. External energy types for active microfluidic applications are critical considerations in this regard because certain physical parameters could be hazardous for biological samples (e.g., cells and proteins). Therefore, thermal field applications are restricted to manipulation of particles that are not damaged by high-temperature gradients. In addition to single methods for particle separation, cascade systems that include more than one particle separation method, are also realistic [103].

The acoustic wave-material interaction causes a change in the wavelength of the propagating wave. Since this change depends on material properties, the transducer design is determined by calculating the velocity of the wave through the material and fabricated on a piezoelectric substrate (e.g., LiNbO_3_, LiTaO_3_) using microelectromechanical fabrication methods. Unlike micromixers, acoustic methods in particle manipulations also differ according to the types of used surface acoustic waves (SAW), such as Rayleigh waves, shear horizontal waves, Lamb waves, and Love waves. The aim of acoustic waves is to bring the particles into order instead of subjecting them to the chaotic effects. Generally, depending on the standing acoustic wavelength or standing surface acoustic waves (S-SAW), acoustic pressure nodes are created inside a microfluidic channel by applying resonant tuned RF signal to the metal ports, which are developed on a piezoelectric substrate or embedded in the body and expressed as interdigitated transducers (IDT)—linear, focused, or tilted at various frequencies. These nodes are high acoustic radiation points and can manipulate the particles depending on characteristic properties such as volume, density, and mass. Thus, they separated by changing their trajectories through different microchannel outputs [20].

To diagnose diseases, purification, or enrichment of bioparticles, such as bacteria and tumors, by separating them from healthy particles such as normal cells, is one of the primary diagnostic methods. For these processes, separating particles by acoustic methods is prevalent. Li et al. [104] proposed an acoustic-microfluidic device to separate unlabeled bacteria from human blood samples. Acoustic radiation forces generated from the tilted angle standing surface acoustic wave (taSSAW) field create an acoustic radiation force that allows separation of Escherichia coli bacteria from human red blood cells at a 96% purification rate when analyzed using flow cytometry analysis [104]. Similarly, a piezoelectric transducer was driven by an AC signal with an amplitude of 15  $V_{p-p}$ and a frequency of 1.99 MHz, creating an acoustic field within the microchannel, which was kept constant at 25 degrees [105]. When the mixture of diluted whole blood cells, to which *P. putida* bacteria were added, and buffer solution were exposed to the effect of the acoustic field, blood cells and bacteria could be separated from each other efficiently [105]. Generally, standing surface acoustic waves (S-SAW) are used to separate tumor cells and healthy cells, allowing cancer diagnosis. Dual IDT structures are excited with identical frequency electrical signals, creating standing waves. The pressure nodes having high acoustic radiation forces ($F_{arf}$) that apply forces depending on cell density and sizes [106,107].

In the electrophoresis method with different particle types (i.e., cations in cataphoresis and anions in anaphoresis), charged particles are separated according to their properties [108]. In contrast, the electrical properties of the particles are redundant in dielectrophoretic-based particle separation [109]. The electric field between two symmetrical or asymmetrical parallel plates results in separation by inducing a force on particles according to their ionicity and polarizability [13,71,110,111,112,113,114,115].

Micro or submicron magnetic particles can be manipulated by creating a magnetic field gradient using oscillations of permanent magnets and electricity-driven coils. This process is a suitable separation method for particles used in biomedical and other fields. The magnetic sensitivity of cells is enhanced by magnetic nanoparticles that are separated from diluted blood using a magnetic field [116]. A similar process can separate white and red blood cells from the blood. Permanent magnets exert positive and negative magnetophoretic forces on RBCs and WBCs. Two cladding streams containing blood plasma condense the cells in the magnetophoretic field. It is possible to separate cells using a magnetic field due to their different properties [117]. As in cells, particles with magnetic properties could be separated using the magnetic field effect [70,118,119,120,121].

#### 2.2.3. Focusing, Sorting, and Enrichment

**Figure 10:**
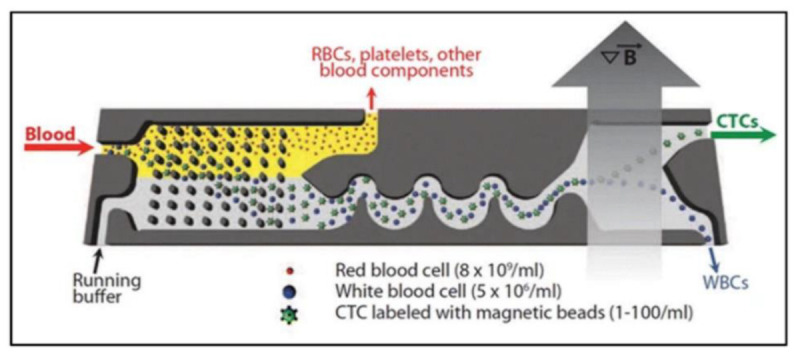
A view of particle sorting mechanism of white blood cells, red blood cells, and circulating tumor cells (CTCs) [128], Copyright 2018, Springer Open.

As opposed to separation, sorting of particles via particle focusing allows accumulation of particles to a point or straight line in active microfluidic applications under the influence of external forces. Focusing phenomena can support the separation of two distinct particle types with different sizes. By taking advantage of the size differences of the particles, large and small particles are subjected to greater or lesser force by the focused acoustic pressure point [122]. In acoustic separations, a pair of electrodes is slotted vertically on two sides of the microchannel, and a surface acoustic wave moves towards the microchannel [123]. Acoustic waves radiated from two opposite directions interfere with each other and generate standing waves that create pressure points at specific points depending on the wavelength inside the linear microchannel [124]. As in acoustic-based resonators and electric field manipulators [7,125], magnetic field-based manipulators [126,127] and optical field-based transducers [8,128] could generate gradients inside the microchannel to manipulate microparticles or nanoparticles for focus in a specific point, sorting in a specific path, and purifying to increase the density inside a content to be used, Figure 10.

#### 2.2.4. Particle Trapping

**Figure 11:**
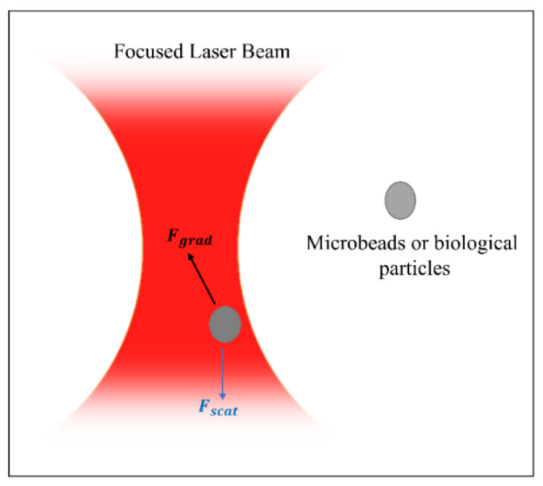
Visualization of single-beam gradient force trapping and forces and the effects of the resulting forces on a particle.

Particle trapping is an extraordinary method actively used in biomedical applications by creating physical tweezers depending on the acoustic field, electric field, magnetic field, optical field, or thermal field as a driving force in every particle manipulation method. Standing waves created by two identical interdigitated transducers are critical in acoustic method for creating a high-pressure point known as a tweezer that can trap different-sized particle, which could be micron- or sub-micron size particles [15,129,130]. Without standing waves, it is possible to confine silica nanoparticles, exosomes, and drugs inside a fluid chamber as rotating droplets for biomedical applications with the help of acoustic radiation force, acoustic microstreaming, and shear stresses [131]. Positive dielectrophoresis (pDEP) and negative dielectrophoresis (nDEP) tapping regions are determined by applied electric field distribution, which could be uniform or non-uniform. The particles, which have a lower polarizability (Re (K) < 0) than the surrounding medium, are driven towards the nDEP and vice versa [9,132]. Magnetic particles move to the low-energy region under the effect of the magnetic field gradient. When the energy distribution is examined, the trajectory of the particle can be predicted and controlled by manipulation of external magnetic energy [133,134]. The Joule heating-induced temperature gradient drives a controllable electric current to manipulate micron-sized particles [11]. In buoyancy-driven convection forces, the particles are pushed through the hot region by a negative Soret coefficient ($S_{T}$), while the opposite is valid for positive $S_{T}$ [12]. Optical tweezing, also known as a single-beam gradient force trap, has the sensitivity of trapping a single molecule or a single nanoparticle and is frequently used in biomedical applications [17]. Depending on the refractive index of particles and surrounding medium, a laser beam creates attractive or repulsive forces [135], which can manipulate even dielectric and absorbing particles at the focal point, Figure 11, which is also known as beam waist [14,19,136].

### 2.3. Summary of Passive and Active Methods in Microfluidics

In summary, passive microfluidics has been used as tools for mixing, particle focusing, or separation. Several techniques, including microfiltration, inertial focusing, secondary flows, DLD, and PFF, were discussed in this section. Also, droplet microfluidics as another area of utilizing passive microfluidics was discussed, and related progress in this area was covered. Since passive microfluidics depends on the channel geometry manipulations of fluids or particles, they can be made with a limited number of parameter (flow rate and fluid viscosity) changes. Still, in active manipulation techniques, the channel geometry, fluid viscosity, the properties of target particles, and the type of external energy source should be considered a priority. Additionally, the generation of external energy related to the frequency and amplitude of the applied signal is also important because the design parameters are tightly correlated. These techniques have their advantages, and are used as is, or will be further developed and used in biomedical applications. Here, micromixers, particle separation, focusing, sorting, enrichment, and particle trapping were mentioned according to the type of external energy, such as acoustic, electrical, magnetic, thermal, pressure fields, and optical, in the active microfluidic section. Lastly, Table 1 summarizes the content of the microfluidic part.

## 3. Fabrication of Microfluidic Devices

Fabrication of micro-sized structures is limited by special requirements such as resolution and difficulty handling small sizes. Therefore, special production techniques have emerged thanks to scientific and technological developments. The leading and current microfabrication methods were reviewed and mentioned in this section. To specify the scope, we examined the fabrication techniques in general in three parts. The first category includes molding, the other three-dimensional printing, and the last category includes nanoimprinting lithography and etching techniques.

### 3.1. Molding

#### 3.1.1. Replica Molding

Replica molding, or soft lithography, is a very common method for fabricating biomedical microfluidic devices [25,137,138,139,140]. Optimized steps exist for the fabrication of silicon molds [2,6]. The negative photoresist using photolithography is patterned on the silicon wafers. SU-8 is usually selected as the photoresist due to its high resolution, mold durability, and capacity for high aspect ratios [141]. The first step is coating the photoresist on the silicon substrate via a spin coater at the corresponding speed for the desired thickness. Then, the wafer is exposed to UV light using a Mask Aligner UV-Lithography device through the previously designed photomask. The next step is to immerse the sample in a developer to remove the unexposed area. In this step, the silicon mold or SU-8 master is ready. The polydimethylsiloxane (PDMS) prepolymer base and curing agent are two chemicals to mix at a 10:1 ratio, and they are then poured over the SU-8 master and placed in a glass petri dish. The PDMS mixture should be degassed before curing in an oven. The PDMS is peeled from the mold and bonded with microscope glass slides by an oxygen plasma device to generate the microchannel. This technique is well established and becoming the standard in the fabrication of microfluidic devices because of its incorporation of a high-resolution, flexible, optically transparent, biocompatible polymer (i.e., PDMS) [137,139,141]. PDMS microfluidic devices control the cell physicochemical environment by adjusting flow conditions [142]. The limitation of soft lithography is the need for a cleanroom facility, which makes this method costly. Also, the molecule absorption of PDMS might influence the cellular response [143,144].

#### 3.1.2. Injection Molding

Injection molding is very attractive for fabrication of microfluidics due to its high-throughput, cost efficiency, and high accuracy [70,137]. This commercially popular method is compatible with a wide range of available thermoplastics and requires a small number of steps [137,145]. First, the used thermoplastic is melted in a compressible chamber, and two sides of the mold are compressed to form the mold cavity. After the mold has cooled down, the cast object is removed. The mold insert techniques and materials depend on the required production of the mold. Lee et al. [146] discussed advantages and limitations of rapid injection molding and provided design recommendations to successfully utilize this method for microscale cell-based assay development. Convery et al. [147] showed that inlays for injection molding could be three-dimensionally printed. Generally, the main drawbacks of microinjection molding are material restrictions related to thermoplastics and mold expensive fabrication and limited resolution [25,137]. In the study of an injection molded microfluidic approach with novel single-cell analysis capabilities were considered [148].

#### 3.1.3. Hot Embossing

The mold shape is transferred to the thermoplastics or polymers at high temperature and pressure during this process. To do so, the thermoplastic film is inserted between two molds, and then both the film and molds are heated under vacuum. Pressing the molds against the softened polymer transfers the mold shape. Finally, the mold is cooled down, and the processed polymer is removed [137,138]. Al-aqbi et al. [149] used hot embossing of PMMA for studies on drug separation from whole blood within three minutes. Using hot embossing, Jiang et al. [150] reported a flexible method for fabricating glass and other amorphous materials for microfluidic channels. Developing a rapid hot embossing device by Jiang et al. [150] evaluated the effects of process parameters (i.e., embossing force, embossing temperature, soaking time, and annealing rate) on the filling behavior of N-BK7 glass in a microhole of silicon carbide mold [151]. In hot embossing, flowing the thermoplastic at a smaller distance causes less stress in the material than injection molding. Restriction in utilized materials and fabrication of complex structures are the limitations of this method [137].

### 3.2. Three-Dimensional Printing

Three-dimensional printing is a relatively new fabrication technique for various microfluidic devices by successive layers of materials. This additive fabrication technology can utilize several materials with different mechanical and physical properties in a single build process [138]. Three-dimensional printing, which can create fine features with lower costs, has some limitations such as low z-resolution, absence of extremely smooth surface finish, limited restricted diversity of transparent materials, and low precision of fabricated hollow and void sections [152]. Various technologies are associated with three-dimensional printing to develop organ-on-chip applications [153]. This section covers four microfluidics fabrication approaches associated with three-dimensional printing: fused deposition modeling, vat polymerization, multi-jet printing, and two-photon polymerization.

#### 3.2.1. Fused Deposition Modeling

Fused deposition modeling (FDM) is an extrusion-based three-dimensional printing method in which a thermoplastic filament is melted, extruded through a nozzle, and solidified by cooling [154]. Even though this technique is simple, effective, and compatible with different materials, the fabricated structures are more sensitive to compressive stress fractures because there is no sufficient fusion between adjacent layers [25]. Moreover, obtaining microchannels with suitable transparency and sizes is challenging [154,155,156]. Quero et al. studied parameters relevant to printing resolution, such as nozzle features, frame and printing bed, layer thickness, and extrusion width. These revealed the potential of FDM for the fabrication of transparent microfluidic devices [155].

#### 3.2.2. Vat Polymerization

Vat polymerization, which involves stereolithography (SLA) and digital light processing (DLP) as rapid prototyping technologies for manufacturing fine features, utilizes UV light to cure the resin and build three-dimensional printed microfluidics. This method consists of three main components: a light source to enable some reactions, light-sensitive precursor materials with reactants (photoinitiators), and a printing platform as a reaction container [138]. A SLA device directed towards a set of coordinates uses a focused light-emitting diode (LED) laser and scanning galvano-mirror to harden the photopolymer. In the DLP method, a build plate moves in a small increment to expose the liquid polymer with a stationary UV light.

The SLA method was used to fabricate three-dimensional printed microneedle arrays with biocompatible resin for transdermal drug delivery [157,158]. The biocompatibility of commercially available photopolymers was also studied for SLA [159,160]. In a smartphone-based detection study, the DLP technique was proper to develop a paper-based microfluidic analysis device for simultaneous detection of multiple biomarkers [161].

#### 3.2.3. Multi-Jet Printing

This three-dimensional printing technique, commercially known as Polyjet, enables manufacturing of high-accuracy microfluidic devices with various materials. A photosensitive resin is ejected as a droplet from an inkjet printhead and then hardened by a light source attached to the inkjet printhead [137]. Sweet et al. used multi-jet printing for fabricating entirely three-dimensionally printed sub-millifluidic and microfluidics finger-powered (electrical power-free) actuators [162]. Another study developed a wearable microfluidic device to collect sweat from the skin [163]. Microfluidic valves as important parts for controlling fluid were also printed with this method [164].

#### 3.2.4. Two-Photon Polymerization

This technology is capable of generating complex and nano-scale structures. A liquid resin volume is exposed to a focused laser, and owing to the nonlinear nature of photoexcitation, some spots are cured, while the remaining liquid is washed away [138]. This high-resolution three-dimensional printing approach was utilized in fabrication of biomimetic placental barrier structures [165], microneedle arrays [166], transparent fused silica glass microstructures [167], and coaxial lamination mixer [168].

### 3.3. Other Fabrication Methods

#### 3.3.1. Nanofabrication

Standard photolithography does not have a high resolution because of the higher wavelength of its light source for patterning designs on the substrate. Three methods, including extreme ultraviolet lithography (EUV), electron beam lithography (EBL), and nanoimprint lithography (NIL) enable ultra-small features by wavelength reduction [137]. EUV generates 13 nm light wavelength to expose a specific photoresist on the substrate. EBL exposes an electron resist coating with a high-energy electron beam instead of light. However, these methods are not extensively used in nanofluidic design due to their high costs and limited throughput [169]. NIL, as the specific type of replica molding, has many applications in microfluidics [170]. It comprises a mechanical process, in which a prepared mold pressed into a resist material and resist hardening could be performed through thermal, chemical, or optical methods [137]. In one study, a biological detection chip with polymer nanostructures could be fabricated using NIL. Zhang et al. [171] detected lunger cancer cells with a size of 10–15 µm by taking advantage of polymer nanostructure adhesion to a specific property of cancer cells.

#### 3.3.2. Wet and Dry Etching

These methods are generally used for the fabrication of silicon and glass microfluidic devices. Wet etching is a fast-etching technique, which requires strong chemicals such as hydrofluoric acid. In addition to the safety and environmental issues, wet etching generates an isotropic profile of the etched channels [172]. However, as a highly precise and controllable method, dry etching provides anisotropic profile. This approach has slower etching rates than wet etching. Both techniques have been utilized in fabricating microfluidic devices and biological detection systems [173,174,175].

### 3.4. Summary of Fabrication of Microfluidic Devices

This part comprises an overview for the recently developed and practical fabrication methods of microfluidic devices, which is summarized in Table 2. According to the characteristics of utilized material, the application of device, volume, cost of production, and specific fabrication methods can be adopted.

## 4. Biomedical Applications

### 4.1. Microfluidics in Diagnosis

#### 4.1.1. Cancer Detection

Cancer, which can occur in any tissue, is one of the most common and deadly diseases in the world [176]. The importance of timely diagnosis of cancer is indisputable. Today, methods such as positron emission tomography, magnetic resonance imaging, and computed tomography are used for diagnosing and staging cancer masses [177]. There is certainly a need for novel approaches in the diagnosis and treatment of cancer, as these methods rely on patients’ exposure to high doses of radiation or chemotherapeutics [178]. Microfluidics, which involve miniaturized devices and precision analysis techniques, are promising for biomedical applications such as cell culture, drug delivery, DNA amplification, and point-of-care (POC).

Chemotherapeutics used in cancer treatment often cause many side effects. By attaching any imaging or locating agent to nanoparticles, both treatment and diagnosis can be achieved. Microfluidic systems used for this purpose in this way include theranostic nanoparticles [179]. Theranostic nanoparticles can be used to monitor drug delivery, drug release, efficacy, the determination of cancer stage, and the mediation of drug delivery at the appropriate dose [180]. Nanocarriers loaded with chemotherapeutics cause the least systemic toxicity while delivering the drug to the target tissue. For instance, fluorescent 5-aminolevulinic chitosan nanoparticles, combined with alginate and conjugated with folic acid, were designed for endoscopic detection of colorectal cancer cells. These nanoparticles entered tumor cells via the folate receptor, accumulated protoporphyrin IX in the cell with the 5-aminolevulinic acid released from the lysosome, and, thus, were proved to be an ideal vector for photodynamic detection [181]. Ryu et al. [182] demonstrated that cathepsin B-sensitive fluorogenic peptide probes conjugated to the surface of glycol chitosan nanoparticles could filter metastatic cells from healthy ones in three mouse models. Another research effort includes the case of the use of hyaluronic acid, iron oxide, and homocamptothecin nanoparticles in human squamous cell carcinoma, both in in vitro and in in vivo studies [183]. Baghbani et al. [184] showed that ultrasound-mediated treatment of doxorubicin-loaded alginate-stabilized perfluorohexane nanodroplets caused tumor regression in mice with breast cancer. A study performed a photodynamic therapy system with near infrared/magnetic resonance imaging by loading Fe3O4 nanoparticles onto redox sensitive chlorine-e6 conjugated dextran nanoparticles to identify breast cancer cells [185]. Quantum dots are avant-garde in vivo imaging tools. For example, Shi et al. [186] developed luminescent magnetic graphene oxide quantum dot nanoplatforms to identify HEPG2 hepatocellular carcinoma from infected blood samples. In another study, quantum dots and anti-cancer drugs were loaded together on lipid carriers to feel and treat H22 cancer cells [187].

Microfluidic systems provide models for examining and eliminating essential mechanisms such as apoptosis, drug resistance, invasion, and metastasis in cancer. As an example, Han et al. [188] developed a redox and pH-sensitive system with mesoporous silica nanoparticles loaded with doxorubicin to overcome drug resistance in breast cancer. In another study, a paclitaxel and lonidamine loaded EGFR targeted polymer nanoparticle drug delivery system was developed for the combined treatment of drug-resistant cells in breast cancer [189]. In order to increase apoptosis and reduce drug resistance in lung cancer, an inhalation system containing siRNAs, targeting MRP1 and BCL2 and mesoporous nanoparticles loaded with doxorubicin and cisplatin, was designed [190]. Furthermore, microfluidics also automated tumor cell culture, enabling the creation of multicellular co-cultures and mimicry of cancer tissue with organoids [191]. For example, a multi-organ microfluidic chip mimicking lung cancer is physiologically suitable for recapitulating the metastasis process [192]. Nguyen et al. developed electrical impedance through a three-dimensional matrix microfluidic system to define single cancer cell migration [193]. Apart from these, research efforts examining tumor cell extravasation [194], invasion [195], and blood-tumor barrier models [196], with microfluidic platforms, have also been conducted.

Microfluidic systems are also employed to specify cancer biomarkers such as CTCs, ctDNA, exosomes, ncRNA, and various cellular metabolites or proteins [191]. In addition, routine measurement of biomarkers in small amounts of fluid samples from cancer patients contribute to personalized medicine. As CTCs mostly express epithelial cell adhesion molecules, antibodies on CTC chips were used for their selection from blood [197]. In the following stages, debulking, inertial focusing, and magnetic separation steps were added to this system, which was named as CTC-iChip [198]. Ganesh et al. designed another microfluidic chip based on a ZnO electrode and pH sensors for the isolation of CTCs [199]. The rm chip combined two approaches based on cell size or immunoaffinity with Rhipsalis (Cactaceae)-like hierarchical structures [200]. However, the monolithic CTC-iChip is also noteworthy, which distinguishes CTCs using epitopes such as cytokeratin, HER2, and prostate-specific antigen [201]. In Western blotting with microfluidics, expression in patient-derived CTCs was profiled with an eight-plexed protein panel [202]. Aptamer nanovectors, used in CTC membrane protein profiling, identified different breast cancer subpopulations by multispectral orthogonal surface enhanced Raman spectroscopy analysis [203]. Microfluidic technologies such as acoustic waves [204], oscillating flow [205], Dean vortex flow [206], and cluster-chip [207] that can separate CTCs from blood in a label-free manner are also worth mentioning. Moreover, the immunoaffinity [208,209,210], nanomembrane filter [211], dielectrophoretic system [212], lateral displacement, and acoustic fluid [213] techniques were used to isolate exosomes. In addition, techniques for detecting exosomes include the fluorescence electrochemical technique [214] and mass spectrometry [215]. Among these techniques, the ExoPCD-chip, which combines the isolation and electrochemical analysis of exosomes, and the herringbone chip (HB chip), stand out due to their superior performance [216,217]. The liberated ctDNAs from tumor cells reflect mutation degree and progression of cancer. The microfluidic solid phase extraction (μSPE) device produced by Compos et al. includes the immobilization, extraction, and replication of cfDNA, and it can also be produced as a low cost platform [218]. In order to isolate cfDNA from serum while minimizing degradation, a rapid and automated microfluidic has developed that combines all three process of plasma separation, residual protein lysis, and cfDNA elution [219]. However, the ncRNAs play a regulatory role in tumor progression at the transcriptional and translational level. In this regard, an oil-saturated PDMS microfluidic system with droplet digital PCR was developed for lung cancer miRNA quantification [220]. By the multiplex qRT-PCR method developed on a microfluidic chip, 384 miRNAs, which are important in the diagnosis and prognosis of prostate cancer, could be purified [221]. Protein-structured substances such as growth factors, cytokines, and hormones secreted by cancerous cells, as a result of the increase in proliferation, are ideal diagnostic tools. Researchers designed a microfluidic integrated microarray in a single platform to identify PSA, TNF-α, IL-1β, and IL-6 proteins in serum samples from prostate cancer patients [222]. Fan et al. reported a blood barcode chip integrated microfluidic system that can rapidly measure a wide panel of proteins from blood [223].

#### 4.1.2. Cardiovascular Disease Detection

Cardiovascular diseases (CVDs), such as stroke, coronary artery, and hypertension, arise from dysfunctionality of the heart and its relevant blood vessels. CVDs are a major cause of premature death worldwide. Social, environmental, cardiometabolic, and behavioral risk factors are some leading determinants of CVD [224,225]. However, aging is the essential factor of CVDs due to the induction of oxidative stress, which results in variations in biological reactions and reactive oxygen species (ROS) [226]. Diagnosis of CVD is crucially important to decrease the mortality rates, and several detection techniques depending upon biomarkers or molecular imaging (MOI) are currently applied in clinics. Nevertheless, improvements in the accuracy, sensitivity, and specificity of the current diagnostics for early-stage detections of CVDs are necessary to establish effective diagnostic systems [227]. Microfluidic diagnostic platforms present favorable features such as portability, fast-responsive analysis, and low reagent use to detect CVD biomarkers. For this purpose, microchannels were modified by particular antigens to determine CVD-associated biomarkers, and several studies have been performed [228,229,230,231,232,233]. Plenty of blood-borne biomarkers such as cardiac troponin I (cTnI), fibrinogen, and C-reactive protein (CRP) are associated with CVDs. However, currently used assays for diagnosis are costly, time-inefficient, and susceptible to batch-to-batch changes. Sinha et al. built a portable microfluidic device with the integration of aptamer probes and field-effect transistor (FET) based sensor arrays [234]. The proposed device can identify four CVDs related biomarkers such as CRP, cTnI, fibrinogen, and N-terminal pro-b-type natriuretic peptide (Nt-proBNP) in only five minutes from small volumes of clinical samples and present favorable results for novel POCT of CVDs. Heart failure (HF) is a common CVD, and the changes in the level of NT-proBNP in the blood are related to the diagnosis of HF. However, current clinical CVD detection methods are not precise enough to evaluate severity and progression of HF according to one single cut-off value of the NT-proBNP biomarker, whereas a rising pattern for long time periods could be a signal for HF. Therefore, POC monitoring of NT-proBNP is vital to prevent HF. As an example, Beck et.al. developed a microfluidic biosensorchip to determine changes in the level of NT-proBNP by modification of silver nanoparticles (AgNPs) as a label [235]. For this purpose, laminar flow assay (LFA) and electrochemical analysis were combined by flow injection analysis (FIA) while detecting of antibody modified AgNPs. The developed biosensor allows for precise detection of NT-proBNP from a finger prick sample volume at home with simple use. Acute myocardial infarction (AMI) is an extensively encountered CVD disease that is life-threatening and sometimes challenging to diagnose since the symptoms could be confused with other diseases. For this reason, Yin et al. demonstrated a snail-shaped microfluidic platform to detect myoglobin (Myo), cTnI, and creatine kinase-MB (CK-MB) biomarkers for diagnosis of AMI. They designed a microfluidic chip by utilizing a chemiluminescence (CL) detector and coating the middle of the chip, which has reaction layer based on particular antibodies. Thus, they obtained a POCT candidate which is able to diagnose three AMI-related biomarkers with higher sensitivity and within a short time of period [236].

#### 4.1.3. Respiratory Infection Detection (SARS-CoV-2)

A novel coronavirus disease (COVID-19) was first reported in late 2019 and resulted in the infection of over 66 million individuals approximately within a year after its discovery [237,238]. SARS-CoV-2 is a RNA virus that could quickly spread among individuals in intimate interaction through respiration and develops in certain regions such as the nasal cavity, pharynx, and lower respiratory tract [239]. One of the essential stages in controlling the spread of SARS-CoV-2 is early diagnosis [240]. As a result, researchers have been working to have a rapid, inexpensive, portable, and sensitive alternatives for detection. Microfluidic-based detection strategies have been widely developed for point-of-care COVID-19 disease detection throughout the pandemic. These strategies could be classified according to detection mechanism in microfluidic devices: antigen detection, anti-SARS-CoV-2 antibody detection, and nucleic acid detection [241]. In another investigation, Ho et al. [242] designed a disposable point-of-care digital microfluidic cartridge to detect the N gene in SARS-CoV-2 by utilizing real-time quantitative polymerase chain reaction (qPCR). According to the study, the DMF cartridge demonstrated uniform droplet formation, homogeneous temperature control, and a suitable fluorescence readout, enabling qPCR POC testing. Recently, paper-based microfluidic devices have been also emerging. Akarapipad et al. [243] utilized a paper based-microfluidic device for comfortable and facilitated detection of SARS-CoV-2 from saliva samples. Evaluation of the flow profile allowed for assessing infection status. The change in surface tension and capillary flow velocity resulted within particle-target immunoagglutination through the channel, which was consequently determined using a smartphone. Similarly, Kim et al. [244] introduced airborne droplets that could be trapped directly on a paper microfluidic device without additional apparatus in less than 30 mins, including capture-to-assay time. The working principle was based on the 10% human saliva samples with SARS-CoV-2 sprayed into the air to produce liquid droplets and aerosols. Subsequently, an antibody-conjugated particle was introduced to the paper channel, and the immunoagglutinated particles on the paper microchip were quantified using a smartphone-based fluorescence microscope. Therefore, SARS-CoV-2 could be identified directly from the air with a portable and low-cost approach. Furthermore, the detection of SARS-CoV-2 N protein utilizing a paper channel was reported, which demonstrated a paper-based enzyme-linked immunosorbent assay on the chip and visual detection and sensitivity of the N protein [245].

Qi et al. [246] developed a microfluidic-coupled capacitive sensor for ultratrace nucleocapsid protein detection. The slight change on the microelectrode array surface was recognized using the solid–liquid interface capacitance with a sensitivity of picofarad level. The reaction time of the sensor response from sample to outcome was estimated to be less than 15 s due to adequate microfluidic enrichment, fulfilling the real-time detection need.

### 4.2. Drug Discovery and Delivery

Patients usually ingest drugs for treatment. In the traditional methods, high doses of drugs, high toxicity, and often side effects occur. Drug delivery systems aim to minimize cytotoxicity by increasing bioavailability and specificity. Microfluidic devices can also be platforms for drug delivery that are easy to control, scale, and replicate [247]. Nanotechnological developments facilitate the controlled release and targeted delivery of drugs by encapsulating them [248]. While experimental studies demonstrate the potential of drug delivery systems, it takes a long term to develop the clinical trial with the efficacy and safety standards that patients can use.

Microfluidic systems allow us to control the effectiveness of drug delivery systems. We can roughly divide drug delivery systems into carrier-based and non-carrier-based. Carriers are formed by encapsulating drugs with organic, inorganic and hybrid molecules. Dendrimers, micelles, liposomes, various polymers, and metallic nanostructures are frequently used in drug delivery systems [249]. Drugs that are less soluble in water become more soluble by the conjugation of drug and polymer complexes. However, the effectiveness of nanocarriers varies depending on their size, shape, and physical/chemical properties [179]. These targeted carriers must be biodegradable and compatible, as well as responsive to stimuli [248]. Doxil is the first successful PEGylated liposomal carrier approved by the FDA in 1995 and has fewer side effects and more toxic to tumor cells than doxorubicin [179]. InFed is an iron complex containing dextran and has been used to treat iron deficiency [250]. Another drug–polymer complex is Abraxane, nanoparticles of paclitaxel coated with human albumin [251]. PEGylated lipid nanoprticles also had success in the delivery of RNA therapeutics, such as Onpattro and Comirnaty [252,253]. In addition, various agents can be attached to the polymer backbone or functional side groups that facilitate targeting and imaging of nanoparticles.

Spark microfluidic systems used in the fabrication of particles are divided into single, mixed, and fully aqueous emulsion templates. While traditional methods such as emulsion, dispersion polymerization, and spray drying are less effective in particle production, technologies such as droplet and flow lithography, electrohydrodynamic co-spraying, photolithography, soft lithography-based printing, and micro molding are considered to be more innovative [254]. Each phase in these systems ensures the production of particles in the appropriate size and shape, as well as is the desired physical, chemical, and biological properties. In this way, thy can be used to develop particles with complex structures such as core-shell, multi-core-shell, janus, and porous ones [255]. The formation of particles from monodisperse droplets occurs using various methods such as polymerization, ionic crosslinking, and solvent evaporation [254]. The chemical structure of drug targets must be among already characterized macromolecules such as nucleic acids, enzymes, proteins, and lipids. Thus, microfluidic systems can also be used for new drug discovery [256]. Microfluidic systems not only improve the drug delivery with precise fluid control, but they also provide benefits for testing drugs before clinical use [247]. For the clinical analysis of the drug delivery systems produced in experimental studies, the animal models should be primarily studied. The first limitations in directing drug delivery systems to the desired target are the barriers in the body at the systemic, microenvironmental, and cellular levels [257]. For example, ellipsoids, discoid-shaped nanoparticles, and nanorods adhere better to blood vessels than spheres [258]. Inhalation of nanoparticles allows rapid passage into lung tissue to avoid extravasation [259]. However, mucus barriers can pass smaller particles while larger ones are filtered out. Methods such as receptor-mediated transcytosis and glucose transporters can be used to cross the blood-brain barrier [260]. Oral administration of polymeric nanoparticles was found to be more active with the gastrointestinal tract than normal drugs [261]. Platforms have also been developed that allow the drug to be released only under certain pH and temperature conditions [262]. Enhanced permeability and retention effects in cancer tissues are also utilized for the accumulation of drugs. In addition, negatively charged particles are more difficult to adhere to the cell membrane, while positive ones can cause cytotoxicity in the cell [263]. Preclinical testing of drugs can be achieved by recapitulating the barriers in the body with microfluidic systems such as organ-on-chip and body-on-chip platforms.

Microfluidics allows drug specificity and adjustable doses or combined drug strategies for personalized therapy [257]. Microfluidic devices can be used in disease remodeling and to increase drug accessibility to cancer cells [264]. It is also possible to evaluate them with their biomarker roles [265,266]. For example, graphene oxide nanoflakes have been used in the detection of pancreatic cancer due to their capacity to bind albumin in plasma [267]. The use of magnetically guided or Au nanoparticles is common. The use of photothermal CAR-T cells in solid tumors is also of interest [268]. Microfluidic systems have been also used for approaches such as gene therapy and gene editing [269].

### 4.3. Disease Modeling

Human diseases are controlled by sophisticated mechanisms that are intrinsically difficult to understand since there is a limitation in direct observation of interference of biological molecules. Hence, methods for disease modeling are of considerable interest for understanding disease pathophysiology and the development of advanced therapeutic strategies. The two-dimensional cell culture method has some drawbacks, including the possibility of cell morphology and polarity changes, which might cave to interruption in cellular-extracellular communication [270]. Moreover, the monolayer structure of two-dimensional cell culture leads to unrestricted availability to reach to the optimum medium, oxygen, and signal molecules. Significantly, the accessibility of the nutrients, oxygen, or/and signal molecules for cancer cells in a living organism could be changeable due to the inherent structure of the tumor [271]. On the other hand, three-dimensional cell culture platforms offer an opportunity to investigate complicated interactions by emulating a physiological environment that approximates the in vivo environment observed in patients [272]. The reason for the similarities between responses of animal models and tumor spheroids against drugs could be the increase in cellular interaction via adhesion [273]. Disease-on-chip models, however, attract widespread interest due to their potential emulation of the disease microenvironment, regulatory factors, and physiological circumstances surrounding organs. The shear force applied by the environment, cell patterning, cell–cell communication, and other factors can be controlled for mimicking the organ and relevant diseases [274]. Furthermore, these platforms offer multi-omic analysis and investigation of the primary biophysical and chemical reasons for cancer formation and cellular-extracellular conditional growth microenvironment [275]. In this section of the review, we will discuss the recent applications of three-dimensional culture models as disease-on-a-chip platforms in the study of human diseases, including cancer, neurological, and pulmonary/lung diseases.

#### 4.3.1. Cancer Modeling

The studies on microfluidic cell culture technology in the literature pointed out different aspects of cancer modeling, including cancer cell invasion [276,277,278,279,280,281,282,283], intravasation [284,285], extravasation [286,287,288], and tumor microenvironment modeling [289,290]. Cell invasion refers to cell motility, including attachment, proteolysis, and relocation of cancer cells, which may result in cancer metastasis [291]. Conventional laboratory strategies, mainly two-dimensional approaches, are limited to providing adequate quantitative data, including multifactors for the determination of cell–matrix interaction, cell–cell communication, and cell invasion [292,293,294]. Since multiple factors exist in tumor invasion, finding and distinguishing the function of such environmental factors are required to comprehend the intercellular dynamics of the tumor invasion. One of the significant factors is the interaction between the tumor environmental niche and human immune system. As an example, Surendran et al. [295] emulated the tumor-immune microenvironment (TIME) as a three-dimensional platform, which represented the role of neutrophils along with chemotaxis and neutrophil extracellular traps (NETosis) in the invasion of ovarian tumor cells. In another study on the cancer invasion, Samandari et al. [296] engineered a stand-alone microfluidic gradient generator to characterize transmission of the chemotactic factors over the hydrogel region, which utilized the hydrogel barriers to isolate the cell culture chamber from the signal channels. Moreover, the proposed detachable PDMS microfluidic chip enabled pump free activity and low-pressure operation, thereby preventing potential leakage. Besides, Amirabadi et al. [297] produced a two-layered three-dimensional environment, serving for invasion of different types of breast cancer cells consisting of wild type, mutated, and promoter hypermethylated E-cadherin containing cells. According to the results, MDA-MB-231 cells, as single cells, invaded the matrix more than MCF-7 and CAMA-1, while CAMA-1 cells unitedly invaded less than MCF-7.

The cancer metastasis process could be defined as an intravasation, where transportation of the cancer cell through the blood vessel occurs. Cancer cells tend to intravasate at locations where the shear stress is lower through the vessel [298] and, therefore, trigger formation of angiogenesis-caused capillary branches [299]. Yankaskas et al. [300] displayed shear stress responses of normal and tumor cells throughout the migration to intravasation. Therefore, they utilized a particular molecule, which behaved as fluid shear sensor of the cells. The microfluidic platform modeled the transition from migration to intravasation, where the cells moving through longitudinal channels moved into an orthogonal channel with induced shear stress. However, recapitulating invasion and intravasation at the same time in cancer modeling is compelling due to the complex tumor microenvironment. Nevertheless, Nagaraju and Truonginvasion et al. [301] designed a microfluidic tumor-vascular model, including a three-dimensional tumor, stroma, and vasculogenesis to investigate invasion and intravasation in a single device. Besides, there have been various studies on emulated tumor microenvironments, such as tumor-on-a-chip or cancer-on-a-chip platforms [302,303]. For instance, Chi et al. [304] introduced a three-layered L-TumorChip platform, combining tumor stroma and microvasculature and investigated the effect of different stromal cells on cancer cell development and the stromal effects on drug responses. In another study, Strelez et al. [289] presented the colorectal cancer (CRC) on-a-chip platforms with the facets of CRC, stromal cross-talk, and mechanical force. Moreover, Fridman et al. [305] mimicked the breast tumor microenvironment, where tumor cells, immune cells, and fibroblasts were encapsulated into different hydrogel scaffolds within a microfluidic platform. Similarly, Haque et al. [290] used patient-derived organoids and mimicked pancreatic ductal adenocarcinoma by exhibiting epithelium–stroma communication and controlled the microenvironment-modulating agents in a lab-on-a-chip model.

#### 4.3.2. Neurological Disease Modeling

Microfluidic modeling platforms have been rapidly developed over the past decade, allowing the advancement of in vitro human nervous system modeling and associated disease models. Central nervous system (CNS) modeling involves handling axons, synapses, and neuronal networks, as well as conditional growth in cell culture for mimicking neural diseases such as Parkinson’s disease (PD), Alzheimer’s disease (AD), and multiple sclerosis (MS) [306]. As an example, Virlogeux et al. [307] established a microfluidic model to ascertain Huntington’s disease (HD) corticostriatal network to understand the uncertain role of pre-and postsynaptic neurons during the first stage of the HD development. This study revealed the great importance of the pre-synaptic compartment in HD, especially for the follow-up therapies. Osaki et al. [308] modeled another neurodegenerative disease, Amyotrophic Lateral Sclerosis (ALS), exploiting iPSC-derived skeletal muscle cells and non–ALS patient-derived MNs. This study evaluated muscle contraction and motor neuron viability under mimicked human physiological and pathological conditions. Regarding the peripheral nervous system (PNS) disease, the loss of myelin sheaths could elicit neurological issues. Since the overall process is unclear, it is formidable to come up with auspicious treatment strategies. Hence, Hyung et al. [309] established a microfluidic platform exhibiting the overall mechanism of myelination, demyelination, and remyelination under the favor of cocultured motor neurons and primary Schwann cells. Significantly, the emulated microenvironment enabled the preservation of long-term coculturing over 40 days. Similarly, Dittlau et al. [310] studied the effects of ALS-causing mutations in an in vitro microfluidic model. The results demonstrated that FUS mutations caused by ALS consequently led to poor neurite regeneration over axotomy and neurite outgrowth. They concluded that a selective HDAC6 inhibitor-enhanced neurite outgrowth and regeneration was at play and, therefore, that HDAC6 inhibition could be used to treat ALS.

#### 4.3.3. Pulmonary/Lung Disease Modeling

Lung-on-a-chip platforms seek to model the evaluation of drug toxicity under physiological conditions and to provide technical assistance for drug screening and personalized diagnosis and therapy [311]. Furthermore, various research studies focused on developing lung disease models, such as lung inflammation, injury, and other pulmonary diseases due to the complexity of the lung anatomy and physiology, which involves airway transportation by small units such as bronchi, bronchioles, and alveoli [311], were developed. For instance, Huh et al. [312] investigated the human pulmonary edema on a microfluidic platform, which exhibited the alveolar–capillary interface of the lung. This system consisted of microchannels surrounded by tight layers of human endothelial cells and pulmonary epithelium subjected to air, fluid flow, and cyclic mechanical strain to simulate breathing activity. The other study showed that the pulmonary artery (PA)-on-a-chip platform allowed researchers to investigate pulmonary arterial hypertension (PAH) regarding molecular and functional alterations in pulmonary vascular endothelial and smooth muscle cells against drugs and disease impellers [313]. COPD (chronic obstructive pulmonary disease) is a serious lung illness caused by restricted airways, leading to breathing complications. Although COPD is associated with neutrophil outflow into the airways through chemotactic migration, there is plenty room to improve knowledge about the utilization of neutrophil chemotaxis for the diagnosis of COPD. As an example, Wu et al. [314] constructed a microfluidic system to quantify the neutrophil chemotaxis in sputum samples from COPD patients.

#### 4.3.4. Liver Disease Modeling

Since liver diseases manifest and develop silently, it is vital to immediately take action following a diagnosis [315]. In vitro studies of the pathogenesis of liver disorders benefit from microfluidic disease-on-chip technologies [315]. Numerous liver disease-on-a-chip systems have been introduced, particularly for investigating fatty liver disease. Non-alcoholic fatty liver disease (NAFLD) emerges from lipid deposition in hepatocytes, which could ultimately lead to hepatic carcinoma. Lasli et al. [316] established a NAFLD-on-a-chip model to investigate steatosis, which was composed of spheroids formed inside inverted pyramid-shaped microwells. Moreover, spheroids were formed by coculturing human hepatocellular carcinoma (HepG2) cells and umbilical vein endothelial cells (HUVECs) in microwells. Steatosis progression might lead to inflammation, which is known as steatohepatitis [317]. Wang et al. [318] designed a NAFLD model as a liver-on-a-chip platform using human-induced pluripotent stem cells (hiPSC) cultured within spheroids. The essential pathogenic characteristics of liver organoids were linked to NAFLD that was investigated on-a-chip after induction by free fatty acid. In addition to NAFLD, scientists examined alcoholic liver disease (ALD) by mimicking physiology or anatomy of the liver. For instance, Lee et al. [319] established an ALD model on a chip, which consisted of mono- and co-cultured spheroids. Ethanol-exposed spheroids exhibited different levels of alcoholic injury. Subsequently, the viability, morphology, cytochrome P450 (CYP450) activity, and hepatic functions of spheroids were investigated.

### 4.4. Tissue Engineering

Tissue engineering (TE) aims to regenerate bioengineered tissues based on cellular growth and focuses on biocompatible materials, such as scaffold, to procure proliferation, replacement, or the repairing of damaged tissues [320,321]. The bioengineered scaffolds enhance the transfer of particular cells and growth factors (GFs) through an impaired site of the tissue to promote tissue regeneration [322]. A feasible scaffold must successfully induce the cellular growth—proliferation—which leads to vascularization. The procedure needs to be biodegradable after healing with no toxic effect [323]. Besides, the dynamics and functionality of an extracellular matrix (ECM) of a specified tissue must be mimicked mechanically, biologically, and physically by the scaffold [324]. Although there are already some advancements in functioning by the bioengineered scaffolds, there are still obstacles in the biomimicking of original tissues.

Bioengineered scaffolds are massively developed under the static cell culture environment using supplementing nutrients, which is practically highly limited due to restrictions in cell–cell, cell–ECM interactions, altering the cellular morphology as a result of inadequate replication of the physiological environment [271,324].

ECM dynamics have a dominant effect on the identification and regulation of tissue-specific cellular responses. In addition, the shape the regeneration process, tissue formation, wound healing, and disease progression could also be affected tremendously by ECM. Hence, the platforms that are capable to mimic the dynamics of the main tissue should be used in the fundamental studies on cellular behavior which leads to the advancements in tissue engineering [325]. However, it was shown that the replication of cellular dynamics is insufficient with the use of traditional static culturing techniques [326]. Herein, microfluidic devices introduce a great platform to understand cell–cell and cell–ECM interactions in a precisely controlled microenvironment by manipulation of the cells. Micro perfusion systems used in microfluidic devices can enhance the delivery of the nutrients across cells and subsequently remove the waste from the system by constant flow in microchannels small dimensions. These models are excellent to mimic in vivo cellular reconstructions [327]. The in vitro microfluidic platforms enable the investigation of distinctive biological pathways by mimicking the principal aspects in natural tissues and organs [248,328]. Thus, several applications of microfluidic platforms in tissue engineering were realized and are discussed in this review as two topics—replication of cellular microenvironment and fabrication of biomaterials [329].

#### 4.4.1. Replication of the Cellular Microenvironment

A cellular microenvironment can be formed by dynamic interactions of cells, interstitial fluid, and ECM that vitally affect the cellular process and functioning through physical, biochemical, and physicochemical mechanisms [330,331]. Therefore, it is crucially important to replicate the dynamics of the cellular microenvironment to analyze the phenotypes for disease modelling and therapeutics. Herein, microfluidic platforms are favorable to construct complex biofidelic cell microenvironments by precisely altering the distribution of oxygen and signaling molecules, controlling the mechanotransduction, and presenting a way to combine them with elements to induce the cells electrically, chemically, or mechanically [329]. The cellular behavior depends on the flow. Therefore, one of the particular flow processes observed in the cell microenvironment is the interstitial flow [332]. Interstitial flow (IF) is a one-way transport of fluid through ECM and a signal from the tumors which vitally affects cancer metastasis. Besides, it delivers proteins and soluble reagents through tumor stroma. In a recent study, a microfluidic platform was fabricated that allowed the investigation of activation and differentiation of cancer cells by mimicking the IF of tumor cells and transport of the soluble factors through tumor stroma from donor cells [333]. Biochemical factors also have a major role in the regulation of cell functioning. Zhang et al. investigated the effect of Ca^+2^ and Sr^+2^ metal ions on osteogenic differentiation of mesenchymal stem cells (MSCs) by employing a microfluidic platform [334]. In that study, the Ca^+2^ and Sr^+2^ crosslinked alginate microgels were produced and processed for encapsulation of single MSCs using a microfluidic system to mimic the three-dimensional stem cell microenvironment. In conclusion, they indicated that Ca^+2^ crosslinked alginate hydrogels triggered the osteogenic differentiation by increasing the matrix mineralization. The stiffness of the microenvironment is another essential factor that alters the cell fate and functioning and tissue development [335,336]. In a recent study, the separation of nasopharyngeal carcinoma 43 (NPC43) cells and nasopharyngeal epithelial 460 (NP460) cells were performed by altering the stiffness, number of layers, and dimensions of the cell microenvironment [337]. These alterations caused changes in the migration of both cell types according to separation by a microfluidic platform.

#### 4.4.2. Fabrication of Biomaterials

Biomaterials are the building blocks of tissue engineering that improve the replication of native ECM by inducing the required cellular functioning in injured tissues by utilization of an artificial framework [338,339]. Several techniques have been used to construct engineered biomaterials including particulate-leaching, freeze-drying, electrospinning, rapid prototyping, solvent casting, and microfluidics [340]. Among these techniques, microfluidics has become an advantageous approach for the fabrication of biomaterials as this approach is cost-effective, safe, and manageable [341]. Moreover, employing both fluid dynamics and shaped microchannels would lead to the fabrication of distinctive biomaterial carriers such as nanoparticles, microfibers, and microspheres [342]. In a recent study, Lei et al. have developed a microfluidic platform to prepare magnetic chitosan microspheres (MCMs) to trigger angiogenesis and epithelization for wound healing with antibacterial activity [343]. As a result, the developed microfluidic platform enhanced the efficient fabrication of MCMs with uniform size and shape. In another investigation by Utoh et. al, the microfluidic system was used to fabricate collagen microfibers by fragmentation phenomenon with continuous flow and altered shear stress [344]. Calcium phosphate (CaP) biomaterial is a commonly used material to promote bone regeneration and repair, and as Galván-Chacón et al. demonstrated, a microfluidic system could alter the physical and chemical properties of CaP for superior efficiency [345]. Furthermore, monodispersed CaP microparticles were synthesized in different sizes using droplet microfluidics, which could directly lead to monitoring of the responsive kinetics. The vascularization is one of the obstacles in tissue engineering, and it is considered an essential process to equally distribute the nutrients and oxygen successfully in engineered tissues [346]. In this regard, microfluidic platforms play a crucial role in intensifying the inherent laminar flow and perfusion flow through cells for vasculogenesis and represent a unique system for microvessels perfusion [329,347]. In a recent study, Wang et al. developed a microfluidics-based technique for the fabrication of the endothelized biomimetic microvessels (BMVs) by alginate–collagen composites [348]. The constructed BMVs exhibited a significant perfusion effect that was also able to induce osteogenic differentiation by releasing BMP-2 and PDGF-BB.

### 4.5. Organ-on-a Chip

An organ-on-a-chip (OoC) is an experimental platform used to reproduce human tissue models to investigate pathophysiology of a disease and novel therapeutical approaches. These platforms contain microfluidic channels and engineered tissues having the ability to mimic organ-specific functions [349,350]. The micro scale system replicates better in vivo cell-microenvironment communications in vitro with incorporation of biophysical/biochemical signals, whereas two-dimensional cell culture models cannot. Therefore, OoC platforms can substitute for two-dimensional cell culture and animal models due to ethical concerns and insufficient reproduction of human pathophysiology [351]. During the recent decade, OoC systems have been extensively utilized to replicate physiological microenvironment of several organs such as the gut [352,353,354], heart [355,356,357], liver [358,359,360], bone [361,362,363], kidney [364,365,366], lung [367,368,369] and brain [370,371,372]. In this section, current OoC studies based on the gut, bone, liver, brain, heart, kidney, and lung will be discussed.

#### 4.5.1. Gut-on-a-Chip

**Figure 12:**
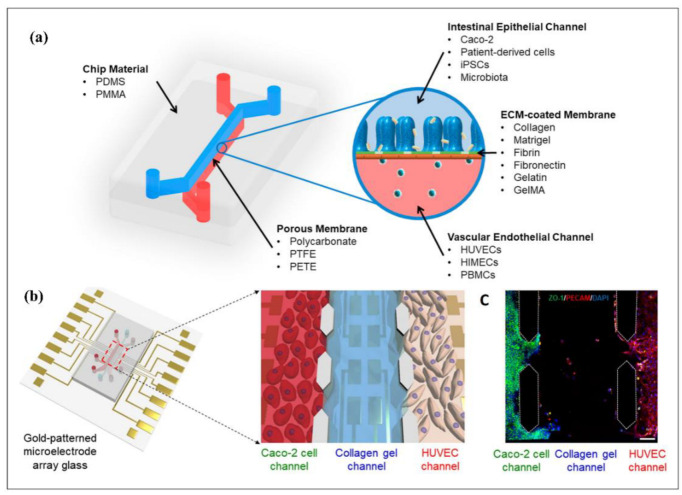
(**a**) A representative model and components of gut-on-a-chip platform. Reprinted with permission from ref. [373], Copyright 2020, Elsevier. (**b**) The human gut-on-a-chip platform was presented by Jeon et al. to reproduce gastrointestinal structure with co-culture of human and microbial cells. The human Caco-2 cells were utilized to form intestinal lumen, whereas HUVECs were employed to establish vascular lumen in right and left channels, respectively. The channels were separated by collagen type I gels, and the continuous flow of medium was enhanced by osmotic pump. **(c)** Immunofluorescence staining results indicated that PECAM-1-positive HUVECs and ZO-1-positive Caco-2 cells were positioned in the left and right channels. Reprinted from ref. [377] (open access).

The essential responsibility of a gut system is nutrient digestion and the restricting of transmission of undesired substances and pathogens for the protection of the body by barrier functioning ability. Nevertheless, the gut is not only vital for the digestive system, but also crucial for the desirable functionality of other organs. Thus, the improper functioning of the gut triggers several diseases [373]. To understand the physiology of the human gut system, animal models and static in vitro models were developed (Figure 12). However, the animal models are not sufficient to mimic the physiology of the human gut, and the static model is not efficient to replicate fluid flow, peristaltic movements, and the villi structures of intestines [374]. Hence, three-dimensional models are required to mimic the gut microenvironment dynamically and to investigate its physiology and pathology properly. Gut-on-a-chip (GoC) platforms are favorable to replicate gut dynamics by consistently perfused microchannels and the utilization of several intestinal cell types to mimic the in vivo morphology of the gut [375]. Maurer et al. developed an intestine-on-a-chip platform to understand microbial interactions in the gut microbiota by replicating the immune tolerance of the intestinal lumen with characteristics of mucosal macrophages and dendritic cells [376]. Hence, efficient investigation of microbial pathogenicity mechanisms under the immunocompetent intestine microenvironment was studied, which can be utilized to explore pathogenic diseases. Jeon et al. designed a gut-on-a-chip platform to study epithelial cell differentiation in vitro [377]. In addition, intestinal epithelial barrier functioning was analyzed with co-culturing of the damaged epithelial layer, and probiotics that consequently promoted healing of barrier functioning were recorded with the assistance of the human microbiome without bacterial overgrowth. In the case of intestinal drug absorption, tissue explants are favorable for drug screening. However, it is not possible to keep the explant tissue alive for a long period in a static environment. Amirabadi and his colleagues developed an intestinal explant barrier chip (IEBC) to analyze intestinal permeability ex vivo [378]. The novel platform incorporated human and porcine intestinal colon tissue explants in separate microchannels to study the intestinal absorption of therapeutics in a dynamic microenvironment with small non-specific binding of therapeutic molecules. The mentioned microfluidic device could be modified for the use in drug screening in other organs such as liver or skin.

#### 4.5.2. Bone-on-a-Chip

**Figure 13:**
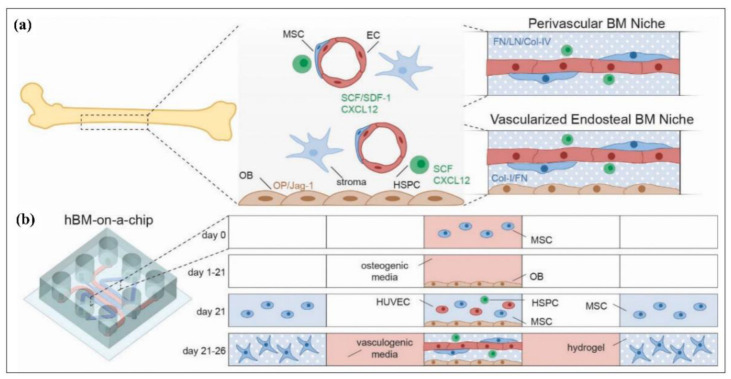
Schematic of human bone marrow-on-a-chip (hBM-on-a-chip) presented by Nelson et al. to investigate hematopoietic stem and progenitor cell behaviour and reaction to pathological stimulus. (**a**) The developed chip can mimic endosteal BM niche and central perivascular BM niche, which are present in long bones. OB = osteoblasts and mineralized bone-like tissue layer; MSC = mesenchymal or marrow stromal cells, including pericytes; stromal cells = other cells of the BM stroma including CXCL12-abundant reticular cells (CAR), matured hematopoietic cells, and adipose cells; HSPC = hematopoietic stem and progenitor cells; FN = fibronectin; LN = laminin, col I and IV = collagen I and collagen IV; OP = osteopontin; Jag-1 = Jagged 1. (**b**) Soft lithography was utilized to fabricate a 5-channel PDMS microfluidic platform. An endosteal layer was formed in the central channel with differentiation of MSCs for 21 days. After this, HSPCs, HUVECs, and MSCs were loaded and seeded on top of the endosteal layer for vasculogenesis. Reprinted with permission from ref. [363], Copyright 2021, Elsevier.

The regulatory effects of the sympathetic nervous system (SNS) on breast cancer bone metastasis were exemplified recently, and Conceição et al. presented a fully humanized metastasis-on-a-chip platform to reproduce the influence of sympathetic stimulus on the cellular interaction between breast cancer cells and bone cells [379]. Three different cell types of osteoclasts, breast cancer cell variants, and sympathetic neurons were cultured in separate chambers, which allowed the dynamic paracrine signaling between the cells. According to the results, the aggression of breast cancer cells increased with the release of paracrine signaling from osteoclasts and sympathetic neurons. The essential role of bone marrow is to establish the hematopoiesis through its endosteal and perivascular niches. Glaser and his team developed a novel microfluidic platform that consisted of two niches of bone marrow separated by vascular network formation [380]. The CD34 + hematopoietic stem cells (HSPC) were cultured and differentiated into mature neutrophils. Thus, the platform allowed one to analyze the stem cell niche and could be utilized for drug screening and modeling of haematological diseases. Multiple myeloma (MM) is an incurable disease which is caused by the accumulation of monoclonal abnormal plasma cells and growth of osteolytic lesions. Nelson et al. [363] demonstrated a similar study based on human bone marrow on chip (Figure 13). However, Sui et al. presented a microfluidic device that replicated the stroma, sinusoidal circulation, and endothelium of the bone marrow microenvironment [381]. Subsequently, the effect of CXCL12- mediated MM cells on the barrier function of endothelial cells was observed, and the device could be used to investigate the spatiotemporal association of cancer cells in the bone marrow sinusoidal microenvironment.

#### 4.5.3. Liver-on-a-Chip

**Figure 14:**
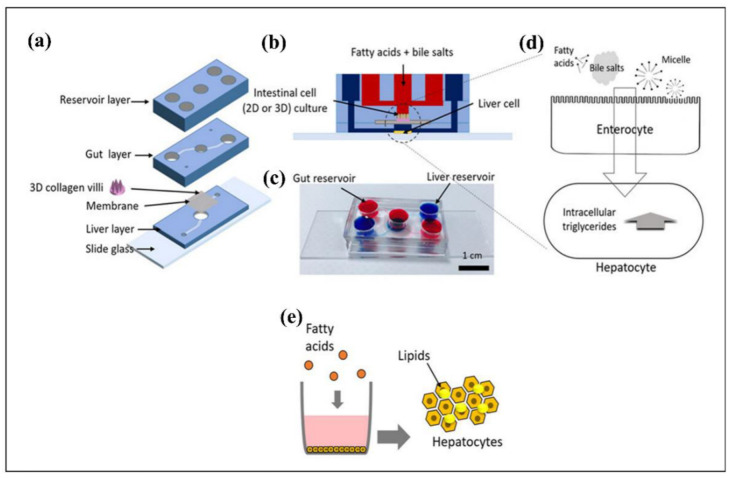
A schematic of gut-liver chip presented by Lee et al. to recapitulate hepatic steatosis. (**a**) Representative figure of gut-liver chip, indicating gut layer top of the membrane and liver layer on the bottom of the membrane. (**b**) Cross-section of the gut-liver chip. (**c**) An image of an assembled gut-liver chip (blue ink indicates the liver part, whereas red ink indicates the gut part). (**d**) This figure shows that absorption of fatty acids by gut cells (enterocytes) and liver cells (hepatocytes) in the gut–liver platform. (**e**) This illustration demonstrates a lipid accumulation experiment in a microwell plate. Cultured hepatocytes (HepG2) in a well plate were exposed to lipid accumulation, and quantification of lipid accumulation was performed. Reprinted with permission from ref. [383], Copyright 2018, Wiley Online Library.

Nonalcoholic fatty liver disease (NAFLD) is the most common liver disease, and its mechanism of progression is still complicated. Du et al. developed a microfluidic-based liver lobule chip (LC), which provided a platform for the co-culturing of hepatic cells and was recruited to investigate NAFLD accurately [360]. In vivo-like liver microtissue was obtained in the LC platform by a dual blood supply from hepatic artery (HA) and hepatic portal vein (PV). The NAFLD was modeled under exposure of nutrient supplies with changes in the lipid zonation for early-stage progression of NAFLD. Obesity is a metabolic disease that emerges with an excessive amount of lipid accumulation, together with escalated inflammation and forms hypertrophic adipocytes. A team led by Leung developed a novel adipose-on-chip (AOC) disease model to reproduce adipose tissue hypertrophy and inflammation under high concentrations of free fatty acid (FFA) [382]. The disease model was replicated by employing oleic acid (OA) and palmitic acid (PA) to initiate inflammation in adipocytes using hypertrophic lipid droplets. The developed model offered a new methodology to investigate obesity-associated metabolic diseases. A study conducted by Lee et al. [383] led to the development of a gut-liver chip to recapitulate hepatic steatosis (Figure 14). In another investigation, the OOC platforms were tested to investigate the toxicological pattern of the therapeutic agent and its metabolites in drug discovery. Soltantabar et al. designed a pumpless heart/liver-on-a-chip (HLC) microfluidic device to explore cardiotoxicity evaluation of doxorubicin (DOX) [384]. The presented HLC platform explained high viability of H9c2 rat cardiomyocytes and HepG2 hepatocellular carcinoma cells. This device was particularly suitable to monitor the damage on heart cells more efficiently compared to three-dimensional static culture. Thus, the developed HLC platform could be a promising tool to investigate cardiotoxicity in the heart.

#### 4.5.4. Brain-on-a-Chip

**Figure 15:**
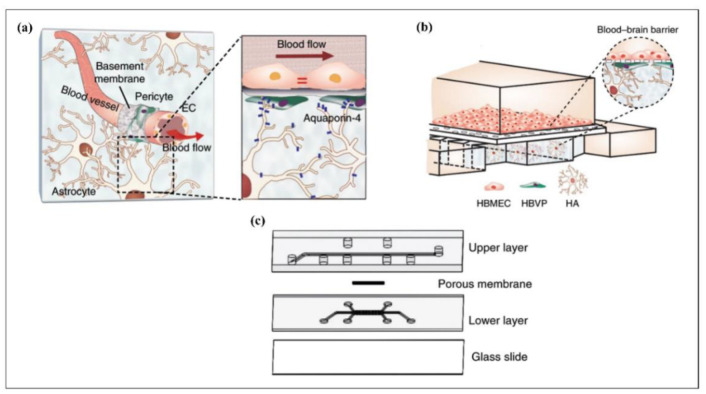
Schematic of human BBB chip presented by Ahn et al. to reproduce the essential structure and function of human BBB and to investigate nanoparticle distribution in the vascular and perivascular parts. (**a**) The figure shows the structure of the BBB composed of endothelial cells (ECs), pericytes, and astrocytes with aquaporin-4 (AQP4) expression. (**b**) Illustration for microengineered human BBB platform. (**c**) Layer-by-layer schematic of developed BBB platform indicates the upper vascular layer, porous membrane, lower perivascular layer, and glass slide. Reprinted from ref. [385] (open access).

Epilepsy is a complex neurologic disease that occurs due to recurrent epileptic seizures. Pelkonen et al. presented a microfluidic platform for epilepsy modeling consisting of a microelectrode array (MEA) that enables one to discriminate seizure-like activity [372]. Human pluripotent stem cells (hPSCs) and differentiated neurons were utilized to form functional neuronal networks, and seizure-like activity was mimicked by kainic acid (KA) treatment on neuronal networks. Several investigations indicated that neurocognitive facilities of the brain can be influenced by the gut environment, and exosomes could also moderate the signaling in the gut–brain axis (GBA). Kim et al. developed a GBA-on chip to investigate gut and brain communication [352]. This microchip was composed of the blood–brain barrier (BBB) and gut barrier, which emulated the co-culture of brain endothelial and gut endothelial cells. The barrier integrity was tested by trans-endothelial/epithelial electrical resistance (TEER), and changes were demonstrated in barriers after lipopolysaccharide (LPS) or butyrate treatment, which eventually induced an inflammatory response in the gut–brain axis and influenced permeability of BBB, respectively. Moreover, as shown in Figure 15, the investigation on the human BBB chip was successful in representing the essential structure and function of the vascular and perivascular parts to study nanoparticle distribution [385].

#### 4.5.5. Heart-on-a-Chip

**Figure 16:**
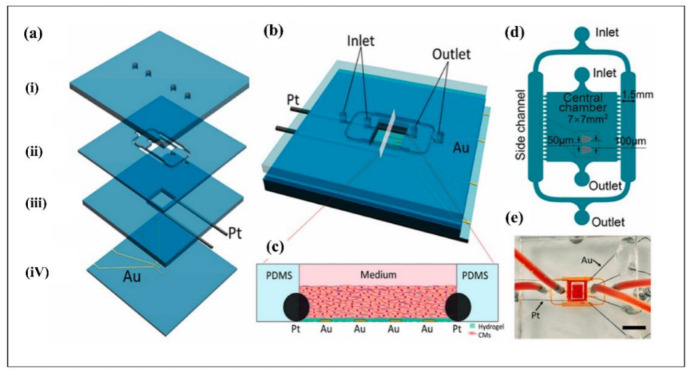
Schematic of heart-on-a-chip device presented by Zhang et al. to investigate in situ electrical stimulation and observation of the function parameters of cardiac tissues. (**a**) Design of the developed heart-on-a-chip platform consisting of four layers: (**i**) top layer—a PDMS cover layer containing 4 inlet/outlet channel; (**ii**) a PDMS channel layer; (**iii**) a PDMS chamber layer inserted with two platinum wire electrodes; and the (**iv**) bottom layer—a glass layer coated by four gold electrodes. (**b**) Three-dimensional illustration of the heart-on-a-chip platform. (**c**) The side view of the schematic demonstrates cardiac tissue in the chamber. (**d**) Magnified sketching of the elaborated design of the PDMS channel, representing the channel layer (ii) in (**a**). (**e**) Picture of the introduced heart-on-a-chip platform. Reprinted with permission from ref. [386], Copyright 2021, Elsevier.

Human-induced pluripotent stem cell (hiPSCs) differentiated cardiomyocytes (CMs) (hiPSCs-CMs) are the key elements to build heart-on-chip platforms. Nevertheless, the immaturity of hiPSCs-CMs, according to the adult myocardium, cause a difficulty in the exact replication of heart physiology and disease. The team led by Zhang et al. developed a novel heart-on-chip platform to overcome the immaturity of hiPSCs-CMs [386] (Figure 16). That microfluidic platform offered a long-term dynamic culture of hiPSCs-CMs, while the real-time recording of hiPSC-CMs under applied electrical stimulation provided the maturation of CMs to replicate native cardiac tissue. The developed heart-on-chip demonstrated favorable results in drug tests and could be proposed as a platform to evaluate drug efficiency and cardiotoxicity.

#### 4.5.6. Kidney-on-a-Chip

**Figure 17:**
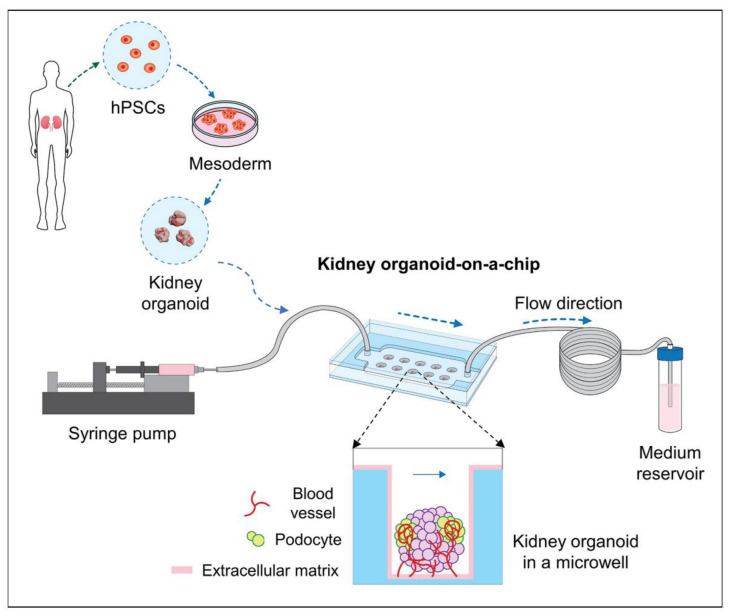
Schematic shows the developed kidney-organoid-a-chip platform introduced by Lee et al. to investigate the biochemical effect on in vitro development of human pluripotent stem cells (hPSCs)-derived human kidney organoids. Controlled shear force and optimized ECM were utilized to explore biochemical effect. Reprinted from ref. [388] (open access).

The glomerulus is the essential element of a kidney, which carries out regular filtration of blood using a capillary network and particular cells known as podocytes. Therefore, mimicking the glomerulus is crucial to investigate kidney physiology and diseases. Roye et al. proposed and demonstrated a personalized glomerulus chip to reproduce glomerulus barrier function utilizing hiPSCs-differentiated nephron progenitor cells and vascular endothelial cells (ECs) from a single patient to obtain a genetically matched tissue profile [387]. In another example, the kidney-organoid-on-a-chip platform was introduced by Lee et al. to investigate the biochemical effect on in vitro development of human pluripotent stem cell (hPSCs)-derived human kidney organoids (Figure 17). In addition, a disease model was also developed to investigate the glomerulus injury, and the results indicated that the glomerulus chip established promising outcomes to replicate a functional glomerulus and glomerulus-related diseases.

#### 4.5.7. Lung-on-a-Chip

**Figure 18:**
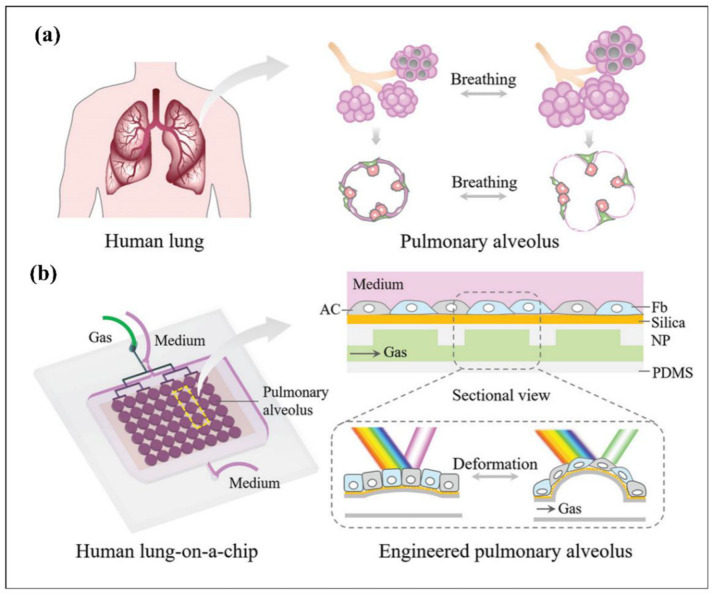
Schematic of lung-on-a-chip platform presented by Zhu et al. to develop favourable biomimetic breathing human lung with microphysiological breathing monitoring. (**a**) Figure of pulmonary alveolus while breathing. (**b**) Design of the developed breathing human-lung-on-a-chip device consisting of an array of pulmonary-alveolus-like structures. Rhythmic stretch during breathing was mimicked by cyclic airflow. Utilization of structural colours was enhanced to visualize breathing process. Fb = fibrinogen; NP = nanoparticle; AC = alveolar cell. Reprinted with permission from ref. [390], Copyright 2022, Wiley Online Library.

Reproducing the air–blood barrier in lung-on-a chip platforms is complicated, and the alveoli network is crucial to mimic physiological properties of the lung in vitro. Zamprogno et al. fabricated a lung-on a chip platform that enabled one to replicate an array of alveoli. This system has an advantage of biodegradability and elasticity in the biological membrane due to the presence of collagen, elastin, and proteins of lung ECM [389]. The platform exhibited an excellent model demonstration for prolonged air–blood barrier functioning using primary human lung endothelial and alveolar epithelial cells and proposed a novel technique to replicate biological barriers of the organs. Furthermore, as shown in Figure 18, the lung-on-a-chip platform studied by Zhu et al. [390] exhibited a favourable biomimetic breathing human lung with microphysiological breathing monitoring.

### 4.6. Microfluidics Biosensors

In recent years, several advantages of microfluidics-based biosensors made them unique methodologies in assay and for the detection of various biological particles. The continues microfluidics-based biosensors provide us with a rapid analysis of biological molecules in a small quantity, with minimum reagent, which consequently generates trivial amounts of byproducts in a single platform with least cost. Biomolecule-based microfluidics biosensors are categorized in four groups based on the type of employed bio elements, including enzymes-based, nanozymes-based, antibody-based, and nucleic acid-based biosensors. Table 3 shows the comparison among them.

#### 4.6.1. Enzyme-Based Microfluidic Biosensors

Enzymes are proteins in nature and are known to enhance the rate of efficiency of a reaction ranging from 105 to 1017 in comparison with non-catalyzers reactions. The biosensor-embedded enzyme generally belongs to the redox enzyme class, which catalyzes oxidation–reduction reactions. Enzymes are perfect biosensors because electrochemical monitoring is typically used to detect their turnover [391].

**Figure 19:**
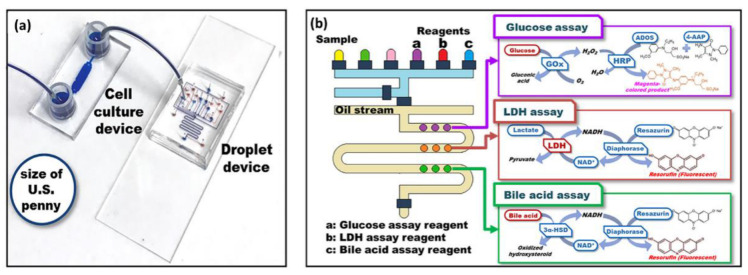
Droplet-based microfluidic platform for cell injury analysis. (**a**) The device was connected to a cell culturing platform for the simultaneous quantification of biochemical analytes. (**b**) Representation of the concept of achieving three different enzymatic assays. Reprinted with permission from [424]. Copyright 2019, American Chemical Society.

Since the first enzymatic biosensor was introduced in 1962 by Clark and Lyon et al. [392], enzymes have been utilized in a diversity of biosensing applications due to their intrinsic functional properties such as high selectivity, biocatalytic activity, and precise enzyme–substrate interactions [393]. By taking advantage of these features, enzyme-based biosensors constitute continuous monitoring and rapid, accurate analysis of several biomarkers [394]. They are usually coupled with microfluidic platforms due to automation, small and stable sensing area, and multiplexed functions [395,396]. In these platforms, reliability, long-term stability, and reusability of enzymes are the main concerns [397]. Enzyme immobilization is one of the most crucial techniques to address these challenges. Immobilization strategies of enzymes onto a surface of a transducer, as well as on the microchannels, have been widely reviewed in the literature [398,399,400,401,402]. Most of the immobilization approaches rely on adsorption, covalent bonding, cross-linking, and entrapment of enzymes. Adsorption is forthright. Physical interactions such as ionic, hydrogen bonding, and Van der Waals forces are responsible for immobilization without disrupting the essential structure of the enzyme [403]. However, covalent bonding is more complicated. It requires strong interaction between surface groups of the enzyme and the surface [404]. Immobilization can be conducted via forming strong covalent bonds between enzymes as well. Cross-linking methods form three-dimensional enzyme complexes by utilizing cross-linking agents for immobilization [401]. Lastly, enzymes can be encapsulated within organic or inorganic polymer matrices to sustain the structural stability of the enzyme and diminish leakage [401,402,403,404,405]. Glucose level measurement is mostly performed by this type of microfluidic biosensor, which were extensively studied in the literature due to the huge demand for diabetes management [406]. In these systems, the widely utilized enzyme is glucose oxidase (GOx) due to its high specificity, low cost, and durability against pH and temperature [406,407]. These advantages make GOx a potent enzyme for microfluidic biosensors to monitor glucose levels in the blood and noninvasive fluids such as saliva, tears, and sweat [408,409,410,411,412]. Recently, Sun et al. [413] developed a microfluidic biosensor for glucose level monitoring from a single drop of any of these noninvasive fluids for the first time. This fully integrated nanoelectronic system was composed of a pump-free, flexible microfluidic enzymatic system (called iez Slice), coupled with a customized reusable potentiostat (called iezBar) for signal acquisition and wireless transference. In this microfluidic platform, to achieve glucose measurement in various raw biofluids including tear, saliva, and sweat, three-dimensional carbonaceous nanosphere network aerogels with hierarchical architectures (3D-CNAs) were used as glucose oxidase electrode substrates due to their higher-level electro-catalytic ability. Moreover, utilization of a microchannel made of high-concentration buffer powder-loaded Kimwipes (HBP-KWs) provided a distinctive stable glucose measurement from tears, sweat, and saliva. Because of the nature of HBP, this microchannel compels biofluids to maintain the same pH and high ionic strength as they do when they flow into it. They noted that, together with the HBP-KWs microchannel, this enzyme-based microfluidic biosensor accurately analyzed glucose from a 0.30 μL sample of raw noninvasive biofluids with a much higher r-value (≥0.96). Monitoring of glucose byproduct lactate level is prominent for athletes and high-performance workers. Additionally, the lactate level is expressed as the best marker of tissue hypoxia [414]. Recently, Shitanda et al. [415] developed a microfluidic sensing system for sweat lactate level tracking. They immobilized the lactate oxidase (LOx) enzyme by a covalent bonding method onto a MgO-templated carbon screen-printed electrode with the aid of an alkene of glycidyl methacrylate (GMA). This electrode configuration supplied a high surface area to acquire a high response readout. Then, this electrode was integrated into a microfluidic platform with eight sweat collecting channels to avoid turbulence and air trapping, and a chamber with a 10 mm radius was introduced. Monitoring the level of another biomarker, urea, provides valuable information about kidney and liver health [416]. Hence, numerous microfluidic platforms, based on the principle of urea hydrolysis by the urease enzyme, exist in the literature [416,417]. Additionally, monitoring creatinine levels for kidney health is among the top applications of enzyme-based biosensors [418]. Tzianni et al. [419] developed a smartphone-coupled paper-based sim card type biosensor for urinary creatinine measurement. Creatinine deiminase enzyme was immobilized onto pH-responsive copolymers PMMA-co-PMAA and demonstrated three conductive electrode configurations. This system was successfully mounted in a sim cardholder. Based on the same principle, various enzyme microfluidic paper-based analytic devices revolutionized the field of microfluidics in biomedical applications. Such an improvement has introduced further sensitivity and selectivity in analytical properties on paper-bound enzyme microfluidics systems [420]. Additionally, enzyme-immobilized papers demonstrate further mechanical and chemical stability due to their collegial effects. Nonetheless, such bindings increase the life span and enzyme stability [421,422]. An enzyme-based paper microfluidics system, μPADs, offers a cellulose matrix which is highly flexible, thin, and cost effective [423]. In addition, the paper has implicit capillary function with high surface-to-volume ratio, enabling the user to load various enzymes and markers. Moreover, the surface of microfluidic cellulose papers is conveniently adjustable for microfluidics channels via two zones attributed to sample and detection [137] (Figure 19).

During the last decade, multiplexed analysis of simultaneous biomarkers in invasive and noninvasive biofluids has accelerated by leveraging microfluidics [425]. For instance, an enzymatic low volume microfluidic platform that simultaneously analyzes a lifestyle biomarker trio—alcohol, glucose, and lactate levels in a low volume of sweat (1–5 μL)—was developed by Bhide et al. [396]. Monitoring free amino acids in the blood serum could give prominent information about the state of several diseases, including cancers [426].

**Figure 20:**
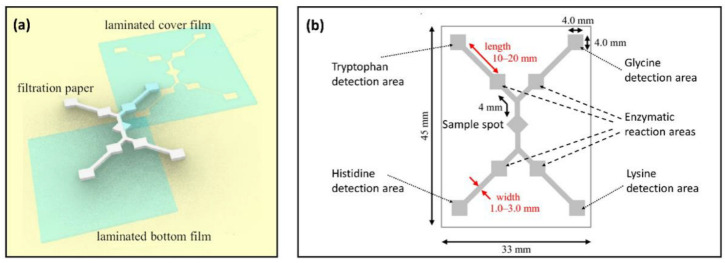
(**a**) Representation of the fabrication of a laminated paper-based analytical device (LPAD) for the simultaneous quantification of tryptophan, glycine, histidine, and lysine levels in the range of a few micromolar to 100 uM by reactions of sample-specific aminoacyl–tRNA synthetases. (**b**) Details of the LPAD. The paper properties and dimensions of channels between detection and reaction areas specify the incubation time for the reaction mixture. Reprinted with permission from [427] (open access).

More recently, Kugimiya et al. [427] developed a laminated paper-based analytical device (LPAD), exploiting an aminoacyl-tRNA synthetase (aaRS) analysis system, to measure histidine, tryptophan, glycine, and lysine levels (Figure 20). It included a sample spot connected to four enzymatic reaction areas, each containing a specific tRNA synthetase for one amino acid type and four detection areas. Properties and dimensions of channels between detection and reaction areas specify the incubation time for the reaction mixture. In the detection zone, the colorimetric signal due to the molybdenum blue reaction was quantified using an image scanner. In another study, ammonia and ethanol concentration in sweat were measured, relying on an enzyme-based colorimetric readout in a multi-layer microfluidic platform. In this platform, super absorbent polymer (SAP) pumps and capillary burst valves were integrated for mixing purposes, as well as to increase the reaction kinetics control [428]. Apart from monitoring the health status of patients, these types of microfluidic sensors are also used to monitor and characterize microfluidic cell culture systems [429]. In an interesting study, Cedillo-Alcantar et al. [424] developed an automated microfluidic platform, relying on droplet generation technology, for this purpose. To emphasize an application, they performed a simultaneous analysis of glucose, bile acid, and lactate dehydrogenase (LDH) of a microfluidic cell culture platform comprising hepatocyte spheroids. To circumvent the problem of decreasing enzyme activities when immobilized, they injected the required enzymes throughout the measurement. With the aid of automated pneumatic valves, first enzymes were mixed with the substrates and then encapsulated in water-in-oil droplets. In each tiny droplet (<0.8 nL), a discrete enzymatic assay took place, and the results were quantified based on colorimetric and fluorescent readouts.

#### 4.6.2. Nanozymes-Based Microfluidic Biosensors

Nanozymes are artificial nanomaterial enzymes that not only mimic the activity of enzymes, but also offer advantages over natural enzymes due to their impressive properties [430]. Compared to natural enzymes, they are easy and inexpensive to produce on a large scale, have a long storage time, and withstand harsh conditions such as high pH and temperatures [430,431,432]. Since they were first introduced in 2007, the use of nanozymes in biosensors instead of enzymes has gained momentum. That year, the interesting enzyme mimetic property of magnetite (Fe_3_O_4_), similar to natural peroxidases, was discovered by Gao et al. [433]. They proposed a new immunoassay in which they used H_2_O_2_Fe_3_O_4_ instead of horseradish peroxidase (HRP) to catalyze the oxidation of various peroxidase substrates. In the following years, other nanomaterials with peroxidase-like properties, such as metal and metal oxides [434], metal–organic frameworks (MOFs), and carbon-based nanomaterials [435] were discovered [431]. Moreover, it was reported that numerous nanomaterials could mimic the activities of other enzymes, including oxidase [436], catalase [437], and superoxide dismutase (SOD) [438,439].

Together with the discovery of enzyme mimetic nanomaterials, their utilization in microfluidic biosensors to perform the function of enzymes has been accelerated. Considering the prominent research area of microfluidic glucose biosensors, nanozymes were effectively used for colorimetric, electrochemical, and fluorescence glucose measurement [440,441,442]. Gomez et al. [441] used a supported metal–organic framework (MOF) to mimic the activity of peroxidase for the first time in a microfluidic paper-based analytical device (μPAD) and measured glucose levels using a small sample volume (10μl) of urine and serum. Another μPAD was designed for simultaneous detection of uric acid and glucose based on peroxidase mimicking platinum nanoparticles (Pt NPs) [443]. Hydrogen peroxide is an important analyte itself and a prominent product or substrate of catalyzed oxidation reactions [444]. Hence, various microfluidic biosensors utilizing nanozymes including Au@PtNP/GO [445], graphene oxide-gold [446], and cerium oxide nanosheets (NSs) [444,447] were developed for H202 measurement. Nanozymes are exploited for point-of-care (POC) cancer diagnosis as well [442,448,449]. More recently Liu et al. [442] developed an electrochemical/visual microfluidic platform for sensitive detection of pheochromocytoma circulating cells (PCC-CTCs) based on the peroxidase mimicking activity of covalent–organic framework-based nanozymes (COF@Pt).

#### 4.6.3. Microfluidics in Antibody Based Biosensing

Antibody-based microfluidic biosensors are based on the immobilization of various monoclonal antibodies as a rapid detection mechanism [450,451]. Using antibodies as biosensors has the advantage that the immunogen could be detected with no prior purification steps [452]. However, recent developments in recombinant technology made it possible to generate the fab fragment as the main antigen binding site for various general antibodies [453]. The limitation of traditional immunoassay techniques has been overcome in combination with microfluidics devices. In the recent pandemic, various portable microfluidics-based immunoassay strips presented accurate, quick, and convenient approaches for the detection of SARS CoV 2 IgG/IgM/antigen using pharyngeal swabs [454]. In this case, the microfluidic immunoassay relies on the interaction of viral protein with immobilized anti-SARS-CoV-2 antibodies on an electrode of sensor with the capacity to detect the change of the electric current. The detectors are generally coated with various materials, such as fluorine-doped tin oxide electrode (FTO) or screen-printed carbon electrode (SPE) or gold nanoparticles AuNPs) or graphene, which consequently act as an indicator for change in conductivity upon antigen/antibody interaction [455,456,457].

**Table 3:** Summary of the advantages and disadvantages in various biosensor-based bio elements [24].

	Enzymes [458,459]	Proteins [460,461]	Nucleic Acid [462,463]	Nanozymes [430,431]
Advantages	High sensitivity and selectivity	Rapid analysis for direct immunoassay	Highly sensitive and selective	Inexpensive to manufacture and easy for large scale production
	Suitable for oxidation and reduction reactions	Suitable for Bio affinity interactions	Ideal for selection of long ranged analytes	pH and temperature stability
		e.g, antibody antigen interaction	Stable, cheap, and easy synthesis	Long storage time
			Potential for modification with labels while retaining same efficacy	
Disadvantages	Possibility of losing their activity upon immobilization	Indirect immunoassay is time consuming and labeling process is costly	Higher toxicity than antibodies	Lower specificity compared to enzymes
	Suitable for small analytes e.g., lactate, urea, glucose	Not ideal for detecting	Faster elimination due to their small size	Biocompatibility and biodegradability concerns
	Sensitive against pH and temperature change	Small targets in both sandwich and direct immunoassay	Weaker binding to analytes	
		Not suitable for redox reactions		

The DNA-based microfluidic biosensors employ amplification of targeted DNA fragments followed by DNA hybridization of obtained sequences of the immobilized complementary target sequence in a single platform. The technique that generally requires separate amplification and base pairing on a gel substrate is integrated into a single tool with a convenient detection method by receiving the relevant change in physiochemical signals [464].

Currently, there are three main microfluidics biosensors developed to detect nucleic acid: PCR, (CRISPR)/clustered regularly interspaced short palindromic repeats, and isothermal amplification [465]. The microfluidics-integrated qPCR method that was reported by several research groups has the advantage of high throughput scheme processing, allowing massive quantitative analysis, including preparation and detection in a single chip platform [466,467]. A recent PCR-based microfluidic system introduced by Cojocaru et al. was a disposable chip functionalized with lyophilized probes and primers, which offered a rapid result (less than 30 mins) for as much as 1.2 μL volume per reaction [468]. In isothermal amplification, the thermocycler, the fundamental part of PCR, is replaced by incubator or water bath-integrated microfluidics devices. Several types of microfluidic-based isothermal amplifications developed, including rolling circle amplification (RCA) [469], recombinase polymerase amplification (RPA) [470], and loop-mediated isothermal amplification (LAMP) [471], which were remarkably efficient and accurate. Ramachandran et al. designed a microfluidics-based CRISPR where the CRISPR–Cas12 enzyme and a guide RNA were introduced to the device to bind to the selected target DNA and cleave it. The active compound then randomly chopped the probed single-stranded DNA labeled fluorophore−quencher. In this method, the CRISPR assay was analyzed by governing the gradients obtained from the change in the electric field and, subsequently, navigated target DNA, reporters, and Cas12–gRNA within the microfluidic device. This technique is known as isotachophoresis (ITP) enhanced microfluidic based CRISPR assay, which can detect the target nucleic acid, RNA, within less than 35 mins [472].

### 4.7. Artificial Cells

The microfluidics systems are also used to provide activated artificial cells via mechanical forces. In this system, stable double emulsion droplets (aqueous/oil/aqueous) are utilized to model mechanosensitive artificial cells. The microfluidics device is designed to trap such mixed drops in the form of the developed chamber, which implies pressure and target simultaneously. Such pressure is accompanied by a temporary raise and perpetual drops in the oil thickness. Therefore, the group observed consequent calcium ion influx due to activated artificial cell activity which was caused by diluted oil drops [473].

### 4.8. Microfluidics and Cryopreservation

Cryopreservation is a banking technique that enhances the store of bio-engineered materials under gradual/rapid cooling and dehydration by adding cryoprotective agents (CPA) [474]. The technique is significant in maintaining particular genetic characteristics of the culture at a certain point for the applications in clinics and tissue engineering [475,476]. In the cryopreservation technique, the biological function of living cells is quenched at low temperatures, which procures their long-term preservation [477]. The cryopreservation technique is very prone to induce cell damage due to dehydration, osmotic shock, and formation of ice crystals, thereby decreasing the cell viability [478]. Microfluidic CPA-integrated devices introduced a great advantage of cryopreservation in a single platform. These procedures, such as automated freeze-thawing cycles with potentially adjustable cell concentration in addition to low-CPA vitrification, were demonstrated with a series of functional microtubes in form of microfluidics channels [479]. In the case of fertility preservation, oocyte cryopreservation is a significant process and, as Guo et al. demonstrated, a microfluidic device could be utilized to minimize the osmotic stress injury (OSI) on porcine oocytes during CPA loading and unloading [480]. They indicated that their invented microfluidic system was able to decrease the potential osmotic damage drastically through the sequencing of loading and unloading CPAs and, thus, promotes the developmental capability of oocytes. Embryo vitrification is another vital process in fertility preservation. Tirgar et. al. designed an automatic standalone microfluidic-based cryopreservation system for mouse blastocyte vitrification [481]. This device enhanced, controlled, and offered a continuous CPA loading without damaging the spherical morphology of blastocytes and minimized the shrinkage rate. Moreover, in a recent study, Özsoylu et al. developed a method to preserve the cell-based biosensor chip, which allowed one to preserve adherent cells on sensor surfaces and, therefore, this method was called on-sensor cryopreservation (OSC) [482]. Here, the biosensor surface was modified by polyethylene vinyl acetate (PEVA) electrospun fibers to make the sensor durable for high temperature changes. Then, the microfluidic platform was integrated through the sensor to ensure fast thawing and decreasing thermal mass. The results indicated that the OSC method is an ideal technique for cryopreservation of adherent cells using sensors and, therefore, it is recommended for “ready-to-use on-site” applications.

### 4.9. Summary of Biomedical Applications of Microfluidics Devices

The biomedical application section explained various techniques where microfluidics devices have been employed for diagnosis, therapeutics, and organ modeling. The role of microfluidic devices was discussed in disease detection, drug discovery, disease modeling, tissue engineering, and organ-on-a-chip use. In the following section, the focus of our review was based on the biosensor development of microfluidic devices in recent years. At last, we have highlighted the two futuristic topics on the application of microfluidic devices in artificial cell development and cryogenic applications.

## 5. Conclusions

Over the last few decades, there have been significant advances in microfluidics devices, which are used in various applications, from diagnostics to disease modeling. Due to the use of biocompatible materials in the production of microfluidic (MF) devices and the development of many production techniques, their use in biological applications has become widespread in recent years. Although there are different production techniques, cost varies depending on the resolution and scale. Although some designs are costly, microfluidic devices can be customized into desired shapes and sizes using soft lithography methods. Lab-on-a-chip platforms have been developed because microfluidic devices are easily integrated and can perform different tasks on the same platform to expedite results. These devices can also be created as a cascade system and can perform all experimental tasks that are normally carried out in laboratories using large-scale systems. Microfluidic devices that are not used directly in the diagnostic tasks can contribute indirectly by carrying out auxiliary tasks such as particle separation and fluid mixing. In addition to the utility of microfluidics as convenient diagnostic tools, modern innovations in microfluidic fabrication techniques improve biomedical integration. Microfluidic bio-systems have revolutionized the field of tissue engineering and organ-on-a-chip. Applications of microfluidic systems were initially focused on food and drug testing. Yet, the need for small-scale biomedical tools featuring microfluidics has expanded over time. Many conventional laboratory diagnostic methods, such as RT-PCR and other molecular analysis methods, have rendered microfluidic systems very powerful tools. Microchannel systems that approximate and emulate the physiological microenvironment of tissues in patients using organ-on-a-chip approaches represent exciting new avenues in which significant progress has been made. Other applications, such as DNA detection and protein detection, have resulted in the development and implementation of microfluidics-based sensor kits. Surface acoustic wave (SAW) sensors, which transduce mass and density changes into electrical signals, represent some of the most promising tools for biomarker detection. In conclusion, this review discussed how different techniques can be developed for fabricating microfluidic devices and other biocompatible materials. Microfluidic devices will find more use in biomedical applications in the future and will reduce diagnostic costs and accelerate diagnosis time. At present, many biomedical studies have actively adopted microfluidics based on their effectiveness in manipulating liquids and particles, sensing changes, and the easy-to-use capabilities of microfluidic structures. One of the major challenges in the development of microfluidics devices with biomedical applications is the transition from laboratory environments to real life industrial application and large-scale production for commercialization. The microfluidic chips to be developed must be portable, durable, and user-friendly. In addition, the microfluidics-based platforms for diagnostic purposes must be adaptable and consistent for sufficient clinical trials and home testing and point-of-care testing. Because of the intrinsic advantages of microfluidics, we anticipate that microfluidics will dominate the biomedical device sector and provide superior diagnostics at a fraction of the time. The development of various geometries with nano/microelectromechanical fabrication methods, combined with easy integration of many external energy forces, may broadly expand the use of microfluidics in biomedical sciences and the clinical practice in the future. Future advances in image/data processing and machine learning techniques will certainly further amplify the impact of futuristic microfluidic devices.

## Figures and Tables

**Table 1:** Summary of passive and active microfluidics.

Microfluidics													
Passive Microfluidics						Active Microfluidics							
Inertial Micromixers	Sorting, Separation, and Isolation				Droplet Microfluidics	Dynamic Micromixers					Particle Separation	Focusing, Sorting, and Enrichment	Particle Trapping
	Microfiltration	Inertial focusing and secondary flows	Deterministic lateral displacement	Pinch flow fractionation	Microfluidic-based materials production	Acoustic field-driven micromixers	Electric field-driven micromixers	Magnetic field-driven micromixers	Thermal field micromixers	Pressure field micromixers			
Summary of Passive and Active Methods in Microfluidics													

**Table 2:** An outline of microfluidic fabrication methods.

Fabrication of Microfluidic Devices								
Molding			3D Printing				Other Fabrication Methods	
Replica molding	Injection molding	Hot embossing	Fused deposition modeling	Vat polymerization	Multi-jet printing	Two-photon polymerization	Nanofabrication	Wet and dry etching
Summary of Fabrication of Microfluidic Devices								

## Data Availability

Not applicable.

## Supplementary Materials

The following supporting information can be downloaded at: https://www.mdpi.com/article/10.3390/bios12111023/s1, table of content.
