# Supplementary material for: Biomedical Applications of Microfluidic Devices: A Review
Source: Biosensors (Basel). 2022 Nov 16;12(11):1023. doi: 10.3390/bios12111023 (PMC9688231; doi:10.3390/bios12111023)
Supplement: Supplementary file 1 [file biosensors-12-01023-s001.zip › biosensors-1959643-supplementary.pdf]

Abstract ········· 1

1. Introduction ········· 1
2. Microfluidics ········· 2
   2.1. Passive Microfluidics ········· 2
      2.1.1. Inertial Micromixers ········· 2
      2.1.2. Sorting, Separation, and Isolation ········· 3
         a. Microfiltration ········· 3
         b. Inertial Focusing and Secondary Flows ········· 3
         c. Deterministic Lateral Displacement ········· 5
         d. Pinch Flow Fractionation ········· 6
      2.1.3. Droplet Microfluidics ········· 6
         a. Microfluidic-Based Materials Production ········· 7
   2.2. Active Microfluidic Devices ········· 8
      2.2.1. Dynamic Micromixers ········· 9
         a. Acoustic Field-Driven Micromixers ········· 10
         b. Electric Field-Driven Micromixers ········· 11
         c. Magnetic Field-Driven Micromixers ········· 11
         d. Thermal Field Micromixers ········· 12
         e. Pressure Field Micromixers ········· 12
      2.2.2. Particle Separation ········· 12
      2.2.3. Focusing, Sorting, And Enrichment ········· 14
      2.2.4. Particle Trapping ········· 14
   2.3. Summary of Passive and Active Methods in Microfluidics ········· 15
3. Fabrication of Microfluidic Devices ········· 16
   3.1. Molding ········· 16
      3.1.1. Replica Molding ········· 16
      3.1.2. Injection Molding ········· 17
      3.1.3. Hot Embossing ········· 17
   3.2. 3D Printing ········· 17
      3.2.1. Fused Deposition Modeling ········· 17
      3.2.2. Vat Polymerization ········· 18
      3.2.3. Multi-Jet Printing ········· 18
      3.2.4. Two-Photon Polymerization ········· 18
   3.3. Other Fabrication Methods ········· 18
      3.3.1. Nanofabrication ········· 18
      3.3.2. Wet and Dry Etching ········· 19

3.4. Summary of Fabrication of Microfluidic Devices ········ 19
4. Biomedical Applications ········ 19
4.1. Microfluidics in Diagnosis ········ 19
4.1.1. Cancer Detection ········ 19
4.1.2. Cardiovascular Disease Detection ········ 21
4.1.3. Respiratory Infection Detection (SARS-Cov-2 ········ 22
4.2. Drug Discovery and Delivery ········ 22
4.3. Disease Modeling ········ 24
4.3.1. Cancer Modeling ········ 24
4.3.2. Neurological Disease Modeling ········ 25
4.3.3. Pulmonary/Lung Disease Modeling ········ 26
4.3.4. Liver Disease Modeling ········ 26
4.4. Tissue Engineering ········ 26
4.4.1. Replication of The Cellular Microenvironment ········ 27
4.4.2. Fabrication of Biomaterials ········ 28
4.5. Organ-on-a-Chip ········ 28
4.5.1. Gut-on-a-Chip ········ 28
4.5.2. Bone-on-a-Chip ········ 30
4.5.3. Liver-on-a-Chip ········ 31
4.5.4. Brain-on-a-Chip ········ 32
4.5.5. Heart-on-a-Chip ········ 33
4.5.6. Kidney-on-a-Chip ········ 33
4.5.7. Lung-on-a-Chip ········ 34
4.6. Microfluidics Biosensors ········ 35
4.6.1. Enzyme-Based Microfluidic Biosensors ········ 36
4.6.2. Nanozymes-Based Microfluidic Biosensors ········ 38
4.6.3. Microfluidics in Antibody Based Biosensing ········ 39
4.7. Artificial Cells ········ 40
4.8. Microfluidics and Cryopreservation ········ 40
4.9. *Summary of Biomedical Applications of Microfluidics Devices* ········ 41
5. Conclusions ········ 41
